# The Cucurbit[7]Uril‐Based Supramolecular Chemistry for Reversible B/Z‐DNA Transition

**DOI:** 10.1002/advs.201800231

**Published:** 2018-05-15

**Authors:** Shao‐Ru Wang, Jia‐Qi Wang, Guo‐Hua Xu, Lai Wei, Bo‐Shi Fu, Ling‐Yu Wu, Yan‐Yan Song, Xi‐Ran Yang, Conggang Li, Si‐Min Liu, Xiang Zhou

**Affiliations:** ^1^ College of Chemistry and Molecular Sciences Key Laboratory of Biomedical Polymers of Ministry of Education Wuhan University Wuhan 430072 Hubei China; ^2^ Key Laboratory of Magnetic Resonance in Biological Systems State Key Laboratory of Magnetic Resonance and Atomic and Molecular Physics Wuhan Institute of Physics and Mathematics Chinese Academy of Sciences Wuhan 430071 Hubei China; ^3^ College of Chemical Engineering and Technology Wuhan University of Science and Technology Wuhan 430081 Hubei China

**Keywords:** cucurbit[7]uril, reversible B/Z‐DNA transitions, spermine, supramolecular chemistry

## Abstract

As a left‐handed helical structure, Z‐DNA is biologically active and it may be correlated with transcription and genome stability. Until recently, it remained a significant challenge to control the B/Z‐DNA transition under physiological conditions. The current study represents the first to reversibly control B/Z‐DNA transition using cucurbit[7]uril‐based supramolecular approach. It is demonstrated that cucurbit[7]uril can encapsulate the central butanediamine moiety [HN(CH_2_)_4_NH] and reverses Z‐DNA caused by spermine back to B‐DNA. The subsequent treatment with 1‐adamantanamine disassembles the cucurbit[7]uril/spermine complex and readily induces reconversion of B‐ into Z‐DNA. The DNA conformational change is unequivocally demonstrated using different independent methods. Direct evidence for supramolecular interactions involved in DNA conformational changes is further provided. These findings can therefore open a new route to control DNA helical structure in a reversible way.

## 1 Introduction

DNA can form a variety of double‐helical structures depending on the sequence as well as the environment.^1^ Unlike the more common right‐handed B‐DNA, Z‐DNA has a strikingly different, left‐handed helical structure with a zigzagging sugar‐phosphate backbone.^2^ The Z‐DNA formation is prone to occur in sequences with alternating pyrimidine–purine segments,^3^ probably due to the favored *syn*–*anti* alternation of the *N*‐glycosylic conformation.^4^ Z‐DNA is naturally biologically active and it may be correlated with genome stability, gene transcription, and gene expression.^5^ However, Z‐DNA has been difficult to study.^6^ This is probably because the thermodynamically more stable B‐DNA is always favored and Z‐DNA easily undergoes spontaneous transition to B‐DNA.^7^ Hence, a favored condition for B/Z‐DNA transition study is reversibility,^8^ which also will help facilitate understanding biological functions of Z‐DNA. Recently, a variety of external stimuli have been employed for in vitro B/Z‐DNA transition.^8^, ^9^ However, some of these approaches still suffer from losing effectiveness outside the proper pH or temperature range. Therefore, it is important to develop new strategy for reversible B/Z‐DNA transition under physiological pH and salt conditions.

Supramolecular chemistry deals with the study of the molecular association in which the partner molecules recognize each other through host–guest interactions.^10^ It can therefore help understanding the recognition and selectivity for biological systems.^11^ Cucurbit[*n*]uril (CB*n*), a pumpkin‐shaped synthetic macrocycle,^12^ represents an attractive host for supramolecular interactions.^13^ The CB*n* molecule can complex with a range of guests due to its hydrophobic cavities and two hydrophilic carbonylated rims.^14^ Of the known CB*n* homologues, CB7 (Figure S1, Supporting Information) has drawn particular attention because of its high biocompatibility and remarkable solubility in aqueous solution.^15^ Importantly, the CB7‐based host–guest systems have been used for some biological applications,^16^ especially in reversible control of the target's structure and function.^17^ Therefore, supramolecular chemistry shows a great potential for tunable structure control of natural bio‐macromolecules.

**Figure 1:**
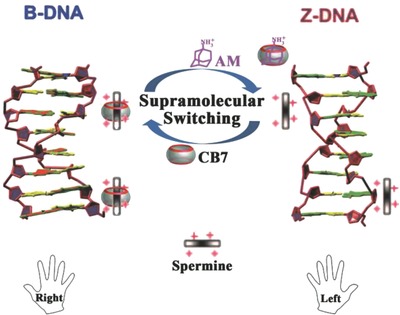
Schematic illustration of the design and workflow. The CB7‐based supramolecular approach is designed for reversible B/Z‐DNA transition.

In the current study, we developed a CB7‐based supramolecular approach for reversible B/Z‐DNA transition (**Figure** **1**). Initially, the spermine [spm in Figure S1 in the Supporting Information, H_2_N(CH_2_)_3_NH(CH_2_)_4_NH(CH_2_)_3_NH_2_] was used to convert B‐ into Z‐DNA. Importantly, CB7 can encapsulate the central butanediamine moiety [HN(CH_2_)_4_NH] to form an inclusion complex (CB7/spm) and reverse Z‐DNA caused by spm back to B‐DNA (right‐to‐left direction in Figure 1). Subsequently, the treatment with 1‐adamantanamine (AM in Figure S1 in the Supporting Information) disassembles the CB7/spm complex and induces the release of active spm. The B‐DNA is thus driven into Z‐DNA again (left‐to‐right direction in Figure 1). We demonstrated the high performance of this “ON/OFF” approach using different DNAs. The conformational change of DNA helical structures has been unequivocally demonstrated using different independent methods. We further provide direct evidence for supramolecular interactions involved in DNA conformational changes. Hence, our findings can open a new route to control DNA helical structure.

## 2 Results

### 2.1 Design of the Supramolecular Approach

The present study is aimed at developing a supramolecular approach capable of manipulating DNA helical structure. An appropriate effector molecule is therefore required. On the basis of previous studies, polyamines show excellent abilities to stabilize Z‐DNA through shielding the negative charge repulsion of sugar–phosphate backbones.^18^ The spm was therefore selected as the candidate. It has been found that CB7 and spm can form an inclusion complex.^19^ This event may cause the change in the steric and electronic effects of the spm guests, which are therefore prevented from gaining access to the DNA phosphate backbone. Hence, CB7 is expected to induce Z–B transition of DNA. AM is known to exhibit an extremely high binding affinity to CB7.^20^ It is therefore expected to reinduce B–Z transition of DNA. Therefore, a plausible supramolecular switch is designed for purpose.

### 2.2 Reversible B/Z‐Transition in a Long Stretch of DNA

**Figure 2:**
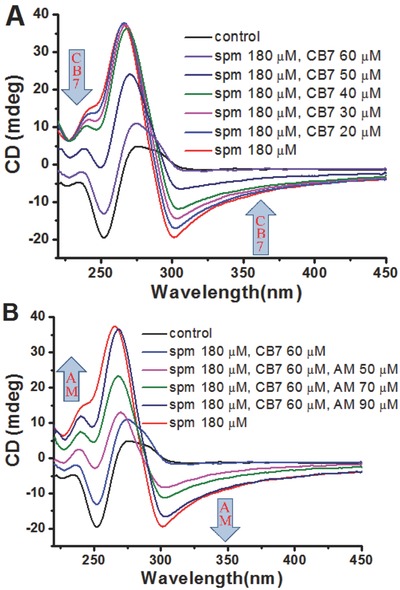
Supramolecular control of B/Z‐transition of polynucleotides. A) The influence of CB7 on DNA helical structure. In this assay, the polyGC (150 × 10^−6^ m) was exposed to 180 × 10^−6^ m spm, and then treated with increasing amounts of CB7 at room temperature for 15 min. B) The influence of AM on DNA helical structure. In this assay, the starting sample (150 × 10^−6^ m polyGC, 180 × 10^−6^ m spm, and 60 × 10^−6^ m CB7) was prepared and subjected to increasing concentrations of AM at room temperature for 15 min. For (A) and (B), the polyGC sample was prepared in 10 × 10^−3^ m Tris‐HCl buffer at pH 7.0 with 50 × 10^−3^ m NaCl. The arrows indicate the change in CD absorbance upon the addition of CB7 or AM.

It has been found that long stretches of alternating purine‐pyrimidine sequences have a tendency to form Z‐DNA.^21^ In our initial study, the commercially available polynucleotide poly(dG‐dC)‐poly(dG‐dC) (polyGC) was chosen as a model sequence. Monitoring the B/Z‐DNA transition has up until now relied almost exclusively on circular dichroism (CD) measurements, since the switch between the right‐ and left‐handed helix results in a very distinctive inversion of the signs of the CD peaks.[[qv: 9b,22]] B‐DNA is usually characterized by a positive long wavelength band(s) at about 260–280 nm and a negative band at around 245 nm, whereas Z‐DNA exhibits an inversion of the spectrum.^23^ Not surprisingly, spm efficiently induces conformational change of polyGC from B‐DNA into Z‐DNA (Figure S2, Supporting Information). Then, we examined the influence of CB7 on the topological states of polyGC in the presence of spm. In accordance with our expectations, CB7 induces significant changes in the CD spectrum (**Figure** **2**A). With addition of increasing amounts of CB7, there is a gradual decrease in the ellipticity around 250 nm along with an increase in that around 290 nm. The appearance of a positive peak at 280 nm and a major negative peak at 250 nm by the addition of 60 × 10^−6^ m CB suggested that the handedness of DNA helix is completely reversed.

Previous studies suggest that the AM molecule can form very stable host–guest complex with CB7.^20^ Therefore, this event is expected to drive the release of free spm and reinduce the transition from B‐ to Z‐DNA. Next, increasing amounts of AM were added to the above two‐component preparation (spm–CB7) and further incubated before CD measurement. Our strategic direction is fully supported by the following results (Figure 2B). At concentrations of AM above 50 × 10^−6^ m, there were evident increases in absorption at 250 nm, indicating the diminishment for CB7 effects. In the presence of 90 × 10^−6^ m AM, the CD spectrum was very close to that observed for the CB7‐free sample, indicating a complete reconversion of Z‐DNA. These results well support the effectiveness of the proposed supramolecular approach.

### 2.3 Reversible B/Z‐Transition in a Short Stretch of DNA

Long stretches of alternating purine‐pyrimidine sequences have been reported to be infrequent in genomes.^24^ Therefore, the B/Z transition in short stretches of DNA is biologically more important than the cooperative transition in long stretches of DNA. Although Z‐DNA formation requires more energy than B‐DNA formation, it can be stable under DNA‐specific conditions such as the presence of negative supercoiling and chemically modified nucleobases.[[qv: 2c,23a,25]] The 2′‐deoxyguanosine (dG in Figure S1 in the Supporting Information) in DNA can be easily oxidized to 8‐oxo‐2′‐deoxyguanosine (8‐oxodG in Figure S1 in the Supporting Information), which represents the most abundant form of oxidative DNA lesion. The 8‐oxodG has been found to facilitate the B‐ to Z‐DNA transition.^26^ Recently, several interesting studies have demonstrated that the formation of 8‐oxodG in gene promoter region can serve as an additional epigenetic mechanism for gene expression control.^27^ It is therefore important to study B/Z transition in short stretches of DNA containing 8‐oxodG.

**Figure 3:**
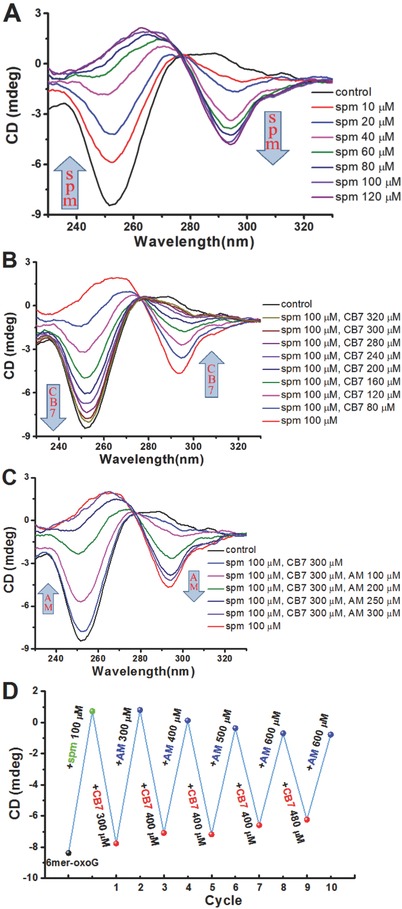
Supramolecular B/Z transition in a short oligonucleotide with 8‐oxodG. A) The influence of spm on DNA helical structure. In this assay, the 6mer‐oxoG1 (50 × 10^−6^ m) was exposed to increasing amounts of spm at room temperature for 15 min. B) The influence of CB7 on DNA helical structure. In this assay, the 6mer‐oxoG1 (50 × 10^−6^ m) was exposed to 100 × 10^−6^ m spm, and then treated with increasing amounts of CB7 at room temperature for 15 min. C) The influence of AM on DNA helical structure. In this assay, the starting sample (50 × 10^−6^ m 6mer‐oxoG1, 100 × 10^−6^ m spm, and 300 × 10^−6^ m CB7) was prepared and subjected to increasing amounts of AM at room temperature for 15 min. D) Repetitive switching of helical structures between B‐DNA and Z‐DNA. In this demonstration, the starting point indicates CD absorbance of the 6mer‐oxoG1 sample (50 × 10^−6^ m) at 253 nm. Subsequently, spm was added, and the resulting solution was analyzed (second point). Next, CB7 and AM were sequentially added to the same sample as indicated. After each addition, the CD absorbance was measured and shown in response to the input (the third to twelfth points). For (A)–(D), the 6mer‐oxoG1 sample was prepared in 10 × 10^−3^ m Tris‐HCl buffer at pH 7.0 with 50 × 10^−3^ m NaCl. The arrows indicate the change in CD absorbance upon the addition of spm, CB7, or AM.

We next investigated the effects of spm on conformation of oligonucleotides containing three contiguous CpG dinucleotides. In these short stretches of DNA, the first or second dG is replaced by 8‐oxodG (6mer‐oxoG1 and 6mer‐oxoG2 in Table S1 in the Supporting Information). The CD spectra of 6mer‐oxoG1 indicated classical B‐DNA conformation. Upon addition of increasing amounts of spm, there was a gradual appearance of a negative peak at ≈290 nm and a positive peak at about 260 nm. From the observations, spm can efficiently induce conformational transition of 6mer‐oxoG1 from B‐ into Z‐DNA (**Figure** **3**A). The treatment with 100 × 10^−6^ m spm resulted in a total conversion of DNA helical structure. In striking contrast, the sequential addition of spm did not significantly influence the conformation of hexamer without 8‐oxodG (6mer‐3G in Table S1 in the Supporting Information), as reflected by very small changes in the shape and intensity of the CD bands (Figure S3, Supporting Information). Moreover, the 6mer‐3G takes the B‐form at spm concentrations up to 2.0 × 10^−3^ m.

Next, we examined whether CB7 can reverse the effects of spm in Z‐DNA induction for 6mer‐oxoG1. Importantly, the sequential addition of CB7 produced evident changes in the shape and intensity of the CD bands (Figure 3B). There was an obvious decrease in absorption at 250 nm and a gradual increase in that at 290 nm as the concentration of CB7 was increased. Specifically, the treatment with 80 × 10^−6^ m CB7 resulted in a significant decrease of Z‐DNA formation, and this effect was more evident with the 160 × 10^−6^ and 240 × 10^−6^ m treatments. Moreover, the treatment with 300 × 10^−6^ m CB7 almost entirely diminished the spm effects on Z‐DNA formation.

We further studied the effects of AM on the double‐helical structures of 6mer‐oxoG1 (Figure 3C). Specifically, B‐ to Z‐DNA transition was obviously enhanced by the 100 × 10^−6^ m AM treatment, and higher levels of enhancement were observed with the 200 × 10^−6^ and 250 × 10^−6^ m treatments. Moreover, the 300 × 10^−6^ m AM treatment largely diminished the CB7 effects and the CD spectrum was very close to that observed for the sample with only spm. From these observations, AM is able to counteract the effects of CB7 on spm and reinduce Z‐DNA in a dose‐dependent manner.

**Figure 4:**
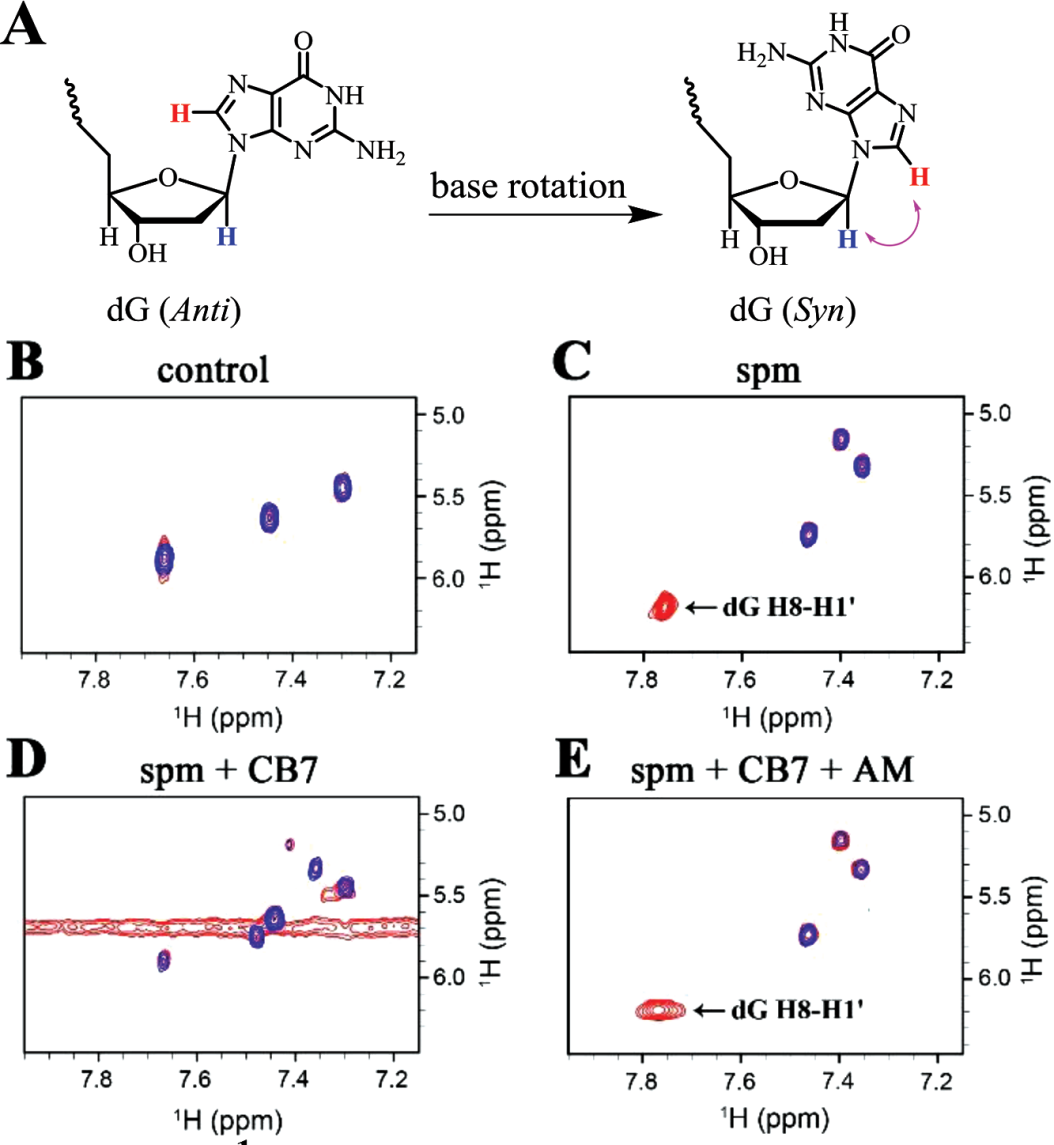
^1^H‐NMR refinement of DNA conformation. A) Schematic illustration of the dG base rotation. The *anti* or *syn* orientation of dG base is demonstrated. B) Acquired spectra in the absence of spm. C) Acquired spectra in the presence of 200 × 10^−6^ m spm. D) Acquired spectra in the presence of 200 × 10^−6^ m spm and 800 × 10^−6^ m CB7. E) Acquired spectra in the presence of 200 × 10^−6^ m spm, 800 × 10^−6^ m CB7, and 800 × 10^−6^ m AM. For (B)–(E), the oligonucleotide 6mer‐oxoG1 (200 × 10^−6^ m) was used. The 2D NOESY and COSY spectra were acquired at 298 K in a buffered solution (2 × 10^−3^ m Tris‐HCl, 50 × 10^−3^ m NaCl, 80% D_2_O/20% H_2_O, pH 7.0). The 150 ms NOESY spectrum (red) is superimposed on COSY spectrum (blue) and selected region is demonstrated. The DNA conformational change is indicated by monitoring the NOE cross‐peak between dG H8 and deoxyribose H1′. The assignment of dG H8–H1′ cross‐peaks relevant to the discussion are labeled.

We next tested whether B/Z‐DNA transition can be controlled for multiple cycles. Hence, 6mer‐oxoG1 was subjected to the spm treatment, followed by sequential addition of CB7 and AM. After each addition, the sample was incubated further before the CD spectrum was recorded. From these observations, the presence of excess CB7 induced broad positive absorbance at 290 nm and strong negative bands at 253 nm, indicating the formation of right‐handed B‐DNA. Strikingly, continual addition of AM produced an inverted CD spectrum with a characteristic negative band at about 290 nm (**Figure** **4**D and Figure S4 (Supporting Information)), indicating the full B‐to‐Z transition by the presence of excess AM. Moreover, the B/Z‐DNA transition of 6mer‐oxoG2 can also be reversibly controlled on demand by adjusting the concentrations of CB7 and AM (Figure S5, Supporting Information).

We next investigated whether CB7 and AM are able to directly influence DNA helical structure. From the observations, the treatment with CB7 did not convert B‐DNA (6mer‐oxoG1, 6mer‐oxoG2, or polyGC) into Z‐DNA in the absence of spm, as reflected by very small variations of the CD spectra (Figures S6A, S7A, and S8A, Supporting Information). Moreover, high concentrations of AM did not markedly affect Z‐DNA structures induced by spm (Figures S6B, S7B, and S8B, Supporting Information). Therefore, these results clearly indicated that the above B/Z‐DNA transition could not be attributed to the direct interaction between the DNA and regulator (CB7 or AM).

### 2.4 ^1^H‐NMR Refinement of the DNA Helicity

To unequivocally verify the helix transitions of 6mer‐oxoG1 between different conformations, 1D and 2D ^1^H‐NMR refinement have been performed. In B‐DNA conformation, the nucleobases are usually in *anti* orientation. However, in left‐handed Z‐DNA, purine bases generally adopt a *syn* orientation.^28^ Therefore, purine bases must rotate with respect to their associated deoxyribose unit along the B‐to‐Z transition (Figure 4A). The ^1^H‐NMR spectral assignment of DNA is usually complicated, especially during conformational transition. However, nonexchangeable proton 2D nuclear Overhauser effect spectroscopy (NOESY) provides a unique method for DNA conformational analysis in solution without prior knowledge of the spectral assignment.^28^, ^29^ Important structural features such as the nucleobase orientation and DNA handedness can therefore be revealed.

As the starting point, 1D and 2D ^1^H‐NMR spectra of 6mer‐oxoG1 were recorded in the absence of spm. Since a full analysis of 2D results is beyond the scope of this study, we have focused on cross sections illustrating correlations between the base aromatic protons (7.95–7.15 ppm) and the deoxyribose H1′ fingerprint protons (6.45–4.90 ppm). Previous research has shown that there is a direct response to the B–Z transition in this region.[[qv: 28,29c]] Figure S9A (Supporting Information) shows expanded plot of the aromatic proton region, in which five major proton resonances are observed. This is consistent with the existence of five bases with nonexchangeable aromatic protons in 6mer‐oxoG1. On the basis of the homonuclear correlation spectroscopy (COSY) spectrum (Figure S9B, Supporting Information), those signals between 7.7 and 7.2 ppm can be readily identified as deoxycytosine (dC in Figure S1 in the Supporting Information) H6/H5 protons since they represent the only aromatic scalar‐coupled protons with 3 bonds apart.[[qv: 29a]] From these observations, the dG H8 proton resonances were assigned vertically between 7.90 and 7.85 ppm.

According to previous studies, the distance between H8 and H1′ in the same dG is 3.7 Å if the glycosidic angle is *anti* or 2.5 Å if it is *syn*, and the distance between cytosine H6 and H5 is 2.5 Å.[[qv: 29a]] Figure S9C (Supporting Information) shows an expansion of NOESY spectrum of 6mer‐oxoG1 demonstrating no cross‐peak to the dG base protons between 7.9 and 7.8 ppm. Figure 4B demonstrates a stacked plot of the NOESY and COSY spectra. On the basis of these results, all NOE signals in the selected window come from dC H6–H5 protons. This phenomenon reveals a longer distance between dG H8–H1′ protons than that between dC H6–H5 protons and strongly suggests the *anti* orientation of the dG base in 6mer‐oxoG1. The helical structure of 6mer‐oxoG1 can therefore be characterized as B‐DNA.

Subsequently, the spm was added to the sample to induce the conformational transition. On the basis of 1D ^1^H‐NMR results (Figure S10, Supporting Information), the chemical shift of dG H8 was observed to gradually shift upfield with increasing amounts of spm. Another interesting finding was that evident shifts were observed for dC aromatic proton resonances. In the presence of 50 × 10^−6^ m spm, dG H8 proton resonances were shifted at a considerable level (≈50%). spm at a higher dose (200 × 10^−6^ m) shifts dG H8 proton resonance to a predominantly new position (Figure S11A, Supporting Information). Next, the COSY spectrum of 6mer‐oxoG1 was recorded in the presence of 200 × 10^−6^ m spm (Figure S11B, Supporting Information). The three major proton resonances between 7.5 and 7.3 ppm were assigned to dC H6/H5 protons due to strong scalar couplings. The NOESY spectrum shows four cross‐peaks in the selected window (Figure S11C, Supporting Information). The stacked plot of the NOESY and COSY spectra displays that only dG H8 (7.75 ppm) and deoxyribose H1′ (6.2 ppm) were connected through space correlations (Figure 4C). This clearly reveals a short distance between dG H8/H1′ protons and strongly suggests the *syn* orientation of the dG bases in 6mer‐oxoG1 in the presence of 200 × 10^−6^ m spm.

Next, increasing amounts of CB7 were added to the sample in the presence of 200 × 10^−6^ m spm. The 1D ^1^H‐NMR results demonstrated that the dG H8 proton signals gradually shifted to the down field along with the addition of CB7 (Figure S12, Supporting Information). Such observed variations show almost completely opposite pattern to that with spm treatment, possibly due to the inclusion of spm into the CB7 cavity. In the presence of 800 × 10^−6^ m CB7, dG H8 proton resonances were almost completely restored to original levels (≈7.9 ppm). We further recorded the 2D spectra of the same sample. The COSY spectrum shows six cross‐peaks, which are correspond to dC H6/H5 peaks over the range 7.7–7.2 ppm in 1D ^1^H‐NMR experiment (Figure S13A,B, Supporting Information). Figure S13C (Supporting Information) shows NOESY spectrum of the same sample. Based on the stacked plot of the NOESY and COSY spectra (Figure 4D), the cross‐peak between dG H8 and deoxyribose H1′ disappeared by the presence of 800 × 10^−6^ m CB7, indicating conformational change of dG base from *syn* to *anti* orientation.

We next tested the effects of AM on the conformation of 6mer‐oxoG1 in the presence of 200 × 10^−6^ m spm and 800 × 10^−6^ m CB7. The 1D ^1^H‐NMR results show upfield chemical shifts for dG H8 proton upon addition of AM (Figure S14, Supporting Information). This trend is in accordance with our expectations. In the presence of 800 × 10^−6^ m AM, dG H8 proton resonance was almost completely shifted and very similar to that with only spm treatment (Figure S15A, Supporting Information). The COSY spectrum of this sample shows three well‐resolved cross‐peaks from dC H6/H5 scalar couplings (Figure S15B, Supporting Information). Importantly, a high‐intensity intraresidual NOE interaction was observed between the more downfield dG H8 signal (≈7.75 ppm) and deoxyribose H1′ resonance (Figure 4E and Figure S15C (Supporting Information)). This phenomenon probably indicates the AM‐induced conformational change of dG base from *anti* to *syn* orientation across glycosidic bond. From these observations, CB7 and AM can provide efficient and effective regulation of DNA helical structure.

### 2.5 Kinetics of B/Z‐DNA Transition

We next studied the kinetics of DNA conformational transitions of 6mer‐oxoG1.^28^ The time‐dependent CD ellipticity changes were monitored immediately after addition of the DNA solution to the amount of a concentrated compound needed to reach the desired concentration. In the first experiment, spm was added to induce B–Z transition of 6mer‐oxoG1. As indicated in Figure S16 (Supporting Information , purple data points), this transition occurs very fast. The CD ellipticity at 253 nm shows a rapid jump followed by a rise which plateaus in a period less than 400 s. The kinetics curve can be well fitted to the monoexponential function (red fitted line), suggesting that the spm‐induced transition from B‐ to Z‐DNA follows the first order.^28^ In the second experiment, CB7 was added to reverse the DNA conformation. Following the addition of CB7, a rapid decrease of absorbance was observed due to DNA conformational change (green data points in Figure S16 in the Supporting Information). This time‐dependent change follows single exponential decay (red fitted line), also indicating the first‐order kinetics. Moreover, after the addition of AM, the absorbance increases logarithmically due to the B‐ to Z‐DNA transition (blue data points and red fitted line in Figure S16 in the Supporting Information). All these results suggest that a two‐state transition model represent an appropriate description for conformational change of 6mer‐oxoG1 in response to different chemical environments.^28^

### 2.6 Thermal Stability and Reversibility Study

**Figure 5:**
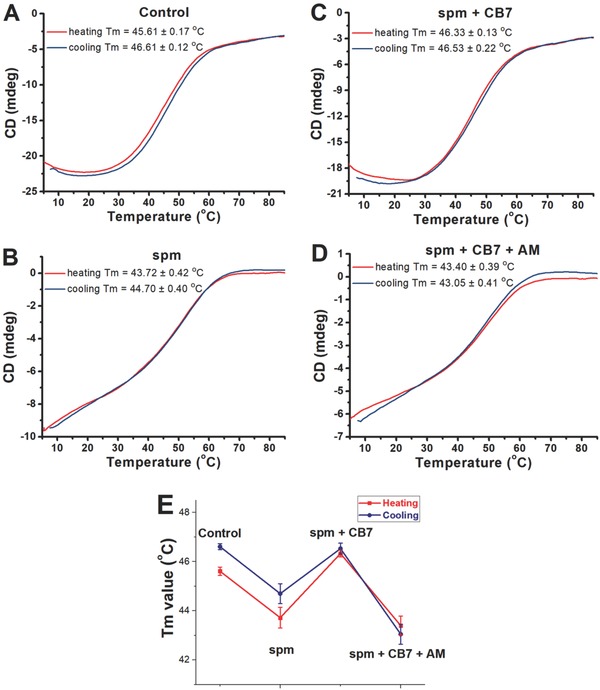
Thermal stability and reversibility of DNA with different handedness. A) Original B‐DNA in the absence of spm. B) Induced Z‐DNA in the presence of 150 × 10^−6^ m spm. C) The B‐DNA in the presence of 150 × 10^−6^ m spm and 800 × 10^−6^ m CB7. D) Reinduced Z‐DNA in the presence of 150 × 10^−6^ m spm, 800 × 10^−6^ m CB7, and 1.0 × 10^−3^ m AM. E) The melting transition for each condition. The *T*_m_ was calculated from the ellipticity‐temperature profile. For (A)–(D), the oligonucleotide 6mer‐oxoG1 (120 × 10^−6^ m) was used. The CD melting and cooling curves were recorded in 10 × 10^−3^ m Tris‐HCl buffer (pH 7.0, 50 × 10^−3^ m NaCl).

We further investigated thermal transition and reversibility of DNA molecules with different handedness by collecting full spectra and monitoring ellipticities at a single wavelength (253 nm) as a function of temperature.^30^ The combined use of these two methods can greatly increase the information about the unfolding and folding characteristics. When spm is absent, heating and cooling experiments demonstrated that thermal denaturation and renaturation of 6mer‐oxoG1 in B‐form are reversible (**Figure** **5**A and Figure S17 (Supporting Information)). The DNA molecules are then exposed to spm, and the resulted Z‐DNA appears to melt directly into single strands rather than transforming into other helical structures prior to melting (Figure 5B and Figure S18A (Supporting Information)). In addition, we observed renaturation of heat‐denatured DNA into the Z‐form by decreasing the temperature (Figure 5B and Figure S18B (Supporting Information)). After subsequent treatment with CB7, the resulting B‐form is thermally stable, with direct melting into single strands at about 46.3 °C (Figure 5C and Figure S19A (Supporting Information)). When the temperature was reduced, again it went back to the B‐form along a reversible way (Figure 5C and Figure S19B (Supporting Information)). Subsequent AM treatment reinduced the B‐to‐Z transition of DNA. No apparent conformational transition, other than denaturation into single strands at around 43.4 °C, has been observed upon heating (Figure 5D and Figure S20A (Supporting Information)). By decreasing the temperature, the single‐stranded DNA appears to refold into the Z‐form (Figure 5D and Figure S20B (Supporting Information)). Importantly, the *T*_m_ values of spm/CB7‐treated DNA molecules were similar to those of untreated DNA molecules. When DNA molecules were subjected to spm or spm/CB7/AM treatment, the *T*_m_ values were significantly decreased, as shown in Figure 5E. This observed tendency is consistent with existing conclusion that DNA molecules are less stable in the left‐handed Z‐from.^7^

### 2.7 Study of Supramolecular Interactions

The ^1^H‐NMR study was next performed to gain more insights into the host–guest chemistry behind reversible B/Z‐DNA transition.[[qv: 16e,19]] Figure S21 (Supporting Information) shows all proton resonances of spm in different chemical environments. There are five chemically inequivalent methylenes, which are labeled with the a, b, c etc. (structure in Figure S21 in the Supporting Information). From our observations, the binding of CB7 to spm induced substantial shifts of all proton resonances. Importantly, the proton resonances of methylenes (a, b, and c) belonging to H_2_N—(CH_2_)_3_—NH— moiety move downfield, indicative of their positioning outside of the CB7 cavity near the portals (blue line in Figure S21 in the Supporting Information). In particular, the largest downfield shift was observed for the proton resonances of “methylene c” attached to secondary amine group and considerably smaller changes in the shifts were observed for the protons on neighboring groups (a and b). However, the proton resonances of methylenes (d and e) belonging to the —HN(CH_2_)_4_NH— moiety moved upfield, indicative of their positioning inside the shielding cavity of CB7. Therefore, it is concluded that CB7 encapsulates the central butanediamine moiety [HN(CH_2_)_4_NH] to form an inclusion complex (CB7/spm).

In a subsequent experiment, evident shifts were observed for the proton resonances of spm methylenes upon addition of AM to the above preparation (CB7–spm) (red line in Figure S21 in the Supporting Information), indicating occurrence of specific decomplexation. This behavior suggests that the binding affinity of CB7 with AM is higher than that with spm. These observations are consistent with those expected from the CD and ^1^H‐NMR results and therefore the host–guest interactions are probably responsible for regulating B/Z‐DNA transition.

**Figure 6:**
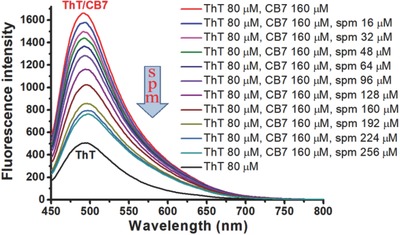
Study of supramolecular interactions between small molecules. The data show fluorescence titrations of the CB7/ThT complex by spm.

To further study supramolecular interaction between CB7 and spm, a competitive dye‐binding assay was performed using the fluorescent probe Thioflavin‐T (ThT).^31^ As shown in Figure S22 (Supporting Information), the aqueous solution of ThT (80 × 10^−6^ m) showed a weak fluorescence band at 493 nm, whereas fluorescence at this wavelength increased with increasing amount of CB7. These observations have established a 2:1 stoichiometry for the (CB7)_2_·ThT complex. Importantly, the emission intensity displayed a gradual decrease by adding spm into the above solution (**Figure** **6** and Figure S23 (Supporting Information)). This expected phenomenon probably indicated the disassembly of the CB7/ThT pair due to the formation of a more stable complex (spm/CB7). By calculating the displacement rate, 1.0 equiv of spm can replace about 50% ThT, and 1.6 equiv can replace more than 80% ThT. These results further demonstrate that the competitive binding takes place directly in this system.

## 3 Discussion

The precise control of DNA structures can help researchers investigate some important biological processes.^32^ In accord with the challenges, previous findings already revealed the potency of host‐guest interactions for this purpose.^33^ There are three major families of DNA helices: A‐DNA, B‐DNA, and Z‐DNA. The A‐and B‐DNA families are right‐handed helices, while the Z‐DNA family has a left‐handed orientation of the helix.[[qv: 2b]] In Z‐DNA, the backbone phosphates are closer to each other than in A‐ and B‐DNA.[[qv: 2a]] In previous researches, different polyamines, such as spm and spermidine, have been used to decrease cross‐groove electrostatic repulsion and induce Z‐form of certain polynucleotides.^34^ However, in these studies, it remains difficult to reverse the effects of polyamines on DNA structure. The potential use of reversible B/Z‐DNA transition under physiological conditions is obvious, for example, it will provide further insights into the functions of Z‐DNA in biology. Hence, this has prompted intensive research to develop novel strategies for reversible control of B/Z‐DNA transition.

The current study represents the first to reversibly control B/Z‐DNA transition using CB7‐based supramolecular approach. Given previous findings that 8‐oxodG, an important oxidative DNA damage, is potentially epigenetic,^27^ the B/Z‐transition of oxidized oligonucleotides has been extensively examined in this study. Our supramolecular strategy is advantageous over other approaches that the transition process could be controlled without adjusting the pH to basic or acidic values. In neutral aqueous solutions, each spm contains four positively charged ammonium groups, which are critical to minimize electrostatic repulsion between the backbone phosphates of Z‐DNA. Interactions between spm and DNA can be readily manipulated utilizing supramolecular competition between spm and AM for binding to CB7. The changes in states of spm resulted in the reversible switching of DNA between different forms. There is a high affinity binding of CB7 to spm (with a binding constant in the order of 10^6^ m ^−1^).^19^ The ^1^H‐NMR study demonstrated that, in the absence of DNA, CB7 can bind spm at the central butanediamine moiety in a 1:1 stoichiometry. However, in the presence of DNA, a several‐fold molar excess of CB7 over spm was required to reverse the Z‐DNA back to B‐DNA.

In the current study, the CD was used as a reference technique to evidence DNA conformational changes.[[qv: 9b,22a]] Our results demonstrate that the structural transition behaviors of DNA with different handedness are quite reversible. Moreover, the ^1^H‐NMR determination elegantly complemented CD measurements and further evidenced the existence of the well‐defined B/Z‐DNA transition. Importantly, COSY experiment was used to group and identify aromatic scalar‐coupled protons on a single base. Moreover, the glycosidic conformation was identified by NOESY experiment on the basis of spatial connectivities between protons coupled by dipolar interaction.[[qv: 28,29c]] By use of these methods, structural information of residues was obtained without giving any information about their position in the sequence or protons on a one sugar. The ^1^H‐NMR experiments reveal that structural transition behaviors are quite reversible. In this context also, it can serve as an interesting starting point for future researchers looking to finish full NMR structural study. Since polyamines are widely dispersed in the cell, the current study further provides new and valuable insight into reversible and accurate control of DNA helical structure in vivo.

## 4 Conclusions

In summary, the present study represents the first to reversibly control B/Z‐DNA transition using CB7‐based supramolecular approach. Using various methods, we provide direct evidence that the CB7‐driven supramolecular competition enables reversible on/off switching of B/Z‐DNA transition. Whether such a strategy is operative in vivo is yet to be understood. We believe that this approach offers a general and facile way toward reversible and accurate control of DNA helical structure.

## 5 Experimental Section

*Chemical Synthesis*: The compound CB7 was synthesized according to the previous literatures.^35^

*Circular Dichroism Study*: The CD experiments were performed at room temperature using a Jasco‐810 spectropolarimeter (Jasco, Easton, MD, USA) equipped with a Peltier temperature controller. All samples were measured in a quartz cell of 1 cm optical path length. The spectra were collected from 340 to 200 nm at a scanning speed of 200 nm min^−1^. The bandwidth was 5 nm and the response time was 2 s. All spectra were baseline‐corrected for signal contributions from the buffer and were the average of three runs.

*Supramolecular Control of B/Z‐DNA Transition*: The desired buffer (10 × 10^−3^ m Tris‐HCl pH 7.0, 50 × 10^−3^ m NaCl) was used throughout the investigation.

*The spm‐Induced Z‐DNA Formation*: The DNA was dissolved to the desired concentration in the desired buffer, heated to 90 °C for 5.0 min, and then cooled slowly to room temperature. The desired spm concentration was obtained by adding appropriate amounts of stock solution, and the mixture was incubated for 15 min at room temperature. Subsequently, the CD spectra were determined and compared to a blank solution.

*The CB7‐Induced Z‐DNA Deformation*: The starting sample was prepared in the desired buffer containing DNA and spm at desired concentrations. After a 15 min incubation at room temperature, CB7 was added sequentially to the starting preparation prior to measurement of CD spectra.

*The AM‐Induced Z‐DNA Reformation*: The starting system was prepared in the desired buffer containing DNA, spm, and CB7 at desired concentrations. The following procedure was similar to the above one wherein AM was added sequentially to the starting preparation prior to measurement of CD spectra.

*^1^H‐NMR Study of DNA Conformation Transition*: The 1D ^1^H‐NMR experiment was routinely performed at 298 K using a Bruker Avance III HD Ascend 850 MHz spectrometer equipped with a 5 mm triple‐resonance (HCN) cryoprobe. The spectra were acquired with a 2 s relaxation delay and 32 transients using watergate pulse sequence with gradients (W5) for water suppression. The MestReNova program was used to process 1D spectra obtained from the original data.

The 2D ^1^H‐NMR experiments were performed at 298 K using a Bruker Avance III HD Ascend 700 MHz spectrometer equipped with a 5 mm triple‐resonance (HCN) cryoprobe. The 2D ^1^H–^1^H COSY and NOESY were collected with spectral widths of 10 ppm and 2048 (40–160 transients) and 120 complex points in the direct and indirect dimensions, respectively. The mixing time for NOESY spectra was 150 ms. The signal assignments were based on the chemical shifts and intensity patterns.

*Thermal Stability and Reversibility Study*: This assay was performed in a Jasco‐810 spectropolarimeter using a water‐jacketed quartz cell. The DNA samples were prepared under the conditions of the experiment shown. The CD spectra were recorded as a function of temperature from 5 to 85 °C.

The CD melting profiles were recorded using a heating rate of 0.2 °C min^−1^ and the absorbance values were collected every 1 °C. The temperature‐dependent CD change was recorded at 253 nm. The melting point corresponded to the mid‐transition temperature.

*Competitive Dye‐Binding Titrations*: The titrations were performed at room temperature in the desired buffer (10 × 10^−3^ m Tris‐HCl and 50 × 10^−3^ m NaCl, pH 7.0).

*The CB7‐Induced Fluorescence Enhancement*: The ThT solution was prepared at a final concentration of 80 × 10^−6^ m. Subsequently, increasing amounts of CB7 (from 0 to 160 × 10^−6^ m) were added sequentially with fast mixing. The fluorescent emission spectra (area: 450–800 nm) were then determined at room temperature using a LS55 fluorescence spectrometer (Perkin‐Elmer Inc., USA). The solution was excited at 421 nm, and a 1 cm path‐length quartz cell was used. Slit width: excitation = 10 nm; emission = 20 nm. All spectra were baseline‐corrected for the signal contributions from the buffer. The plot of emission@493 nm was recorded as a function of the concentration of CB7 added.

*The spm‐Induced Fluorescence Change*: The CB7/ThT complex was prepared in the desired buffer at desired concentrations. The following procedure was similar to the above one wherein spm was added sequentially to the starting preparation prior to fluorescence measurement. The plot of emission@493 nm was recorded as a function of the concentration of spm added.

## Conflict of Interest

The authors declare no conflict of interest.

## Supporting information

Supplementary
