# Supplementary material for: The Cucurbit[7]Uril‐Based Supramolecular Chemistry for Reversible B/Z‐DNA Transition
Source: Adv Sci (Weinh). 2018 May 15;5(7):1800231. doi: 10.1002/advs.201800231 (PMC6051393; doi:10.1002/advs.201800231)
Supplement: Supplementary file 1 — Supplementary [file ADVS-5-1800231-s001.pdf]

Copyright WILEY-VCH Verlag GmbH & Co. KGaA, 69469 Weinheim, Germany, 2018.

ADVANCED SCIENCE
Open Access

Supporting Information

for *Adv. Sci.,* DOI: 10.1002/advs.201800231

The Cucurbit[7]Uril-Based Supramolecular Chemistry for Reversible B/Z-DNA Transition

Shao-Ru Wang, Jia-Qi Wang, Guo-Hua Xu, Lai Wei, Bo-Shi Fu, Ling-Yu Wu, Yan-Yan Song, Xi-Ran Yang, Conggang Li, Si-Min Liu, and Xiang Zhou**

Supporting Information

The cucurbit[7]uril-based supramolecular chemistry for reversible B/Z-DNA transition

Shao-Ru Wang[a,‡,], Jia-Qi Wang[a,‡], Guo-Hua Xu[b,‡], Lai Wei[a], Bo-Shi Fu[a], Ling-Yu Wu[a], Yan-Yan Song[a], Xi-Ran Yang[c], Conggang Li[b], Si-Min Liu[c], Xiang Zhou[a,]**

Table of contents

	General methods and materials
Table S1	Sequences of oligomers used in the current study
Figure S1	Demonstration of chemical structures of spm, CB7, AM, dG, 8-oxodG and dC
Figure S2	The influence of spm on DNA helical structure
Figure S3	The influence of spm on DNA helical structure
Figure S4	Repetitive switching of DNA helical structures between B-form and Z-form
Figure S5	Supramolecular B/Z transition in a short oligonucleotide with 8-oxodG
Figure S6	The influence of different treatments on DNA helical structure
Figure S7	The influence of different treatments on DNA helical structure
Figure S8	The influence of different treatments on DNA helical structure
Figure S9	^{1}H-NMR refinement of DNA conformation in the absence of spm
Figure S10	1D ^{1}H-NMR titration of 6mer-oxoG1 with spm
Figure S11	^{1}H-NMR refinement of DNA conformation in the presence of 200 μM spm
Figure S12	1D ^{1}H-NMR titration of 6mer-oxoG1 with CB7
Figure S13	^{1}H-NMR refinement of DNA conformation in the presence of 200 μM spm and 800 μM CB7
Figure S14	1D ^{1}H-NMR titration of 6mer-oxoG1 with AM
Figure S15	^{1}H-NMR refinement of DNA conformation in the presence of spm, CB7 and AM
Figure S16	The B/Z-DNA transition kinetics

Figure S17	Thermal denaturation and renaturation of B-DNA in the absence of spm
Figure S18	Thermal denaturation and renaturation of Z-DNA in the presence of spm
Figure S19	Thermal denaturation and renaturation of B-DNA in the presence of spm and CB7
Figure S20	Denaturation and renaturation of Z-DNA in the presence of spm, CB7 and AM
Figure S21	^{1}H-NMR analysis of spm in different chemical environments
Figure S22	Fluorescence titrations of ThT by CB7 in aqueous solution
Figure S23	Fluorescence titrations of the CB7/ThT complex by spm in aqueous solution

General methods and materials

Materials

Adamantanamine hydrochloride (CAS# 665-66-7), glycoluril (CAS# 496-46-8), formaldehyde solution (CAS# 50-00-0), Tris(hydroxymethyl)aminomethane (Tris base, CAS# 77-86-1), ThT (CAS# 2390-54-7) and polynucleotide poly(dG-dC)-poly(dG-dC) (polyGC, CAS# 90385-88-9) were purchased from Sigma-Aldrich Inc. (St. Louis, MO, USA), respectively, and used without further purification. The polyGC is a repetitive synthetic double-stranded DNA sequence of up to 800-1000 base pairs in length. The oligonucleotides were obtained from Takara Corporation (Dalian, China). All buffer solutions were prepared with ultrapure water (18.2 Ω·cm, Milli-Q system). The pH was determined using Mettler Toledo™, FE20 FiveEasy™ Benchtop pH Meter (Mettler Toledo, Switzerland). The DNA concentration was quantified using NanoDrop 2000c (Thermo Scientific, USA).

Kinetic studies

The measurement of B/Z-DNA transition kinetics was carried out immediately after addition of the DNA solution to the amount of a concentrated compound needed to reach the desired concentration. The time-dependent CD change was recorded at 253 nm. Given the considerable time scale of the transition, the time necessary to add the DNA solution into the quartz cell and to start the measurement (~3 s) was considered negligible. The CD spectra of the DNA was determined at several time intervals.

^{1}H-NMR study of host-guest interactions

The ^{1}H-NMR spectra were recorded at 298 K using a Bruker Avance III HD Ascend^{TM} 850 MHz spectrometer equipped with a 5 mm triple-resonance (HCN) cryoprobe. The spm (1.0 mg) was dissolved in 240 μL of D_2O and then analysed. Subsequently, CB7 (6.81 mg) was added to the above solution at room temperature and then analysed. Next, AM (0.93 mg) was added to the above CB7-spm preparation and analysed. The signal assignments are based on the chemical shifts and intensity patterns. The MestReNova program was used to process the 1D ^{1}H-NMR spectra obtained from the original data.

Table S1 Sequences of oligomers used in the current study

Oligomer	Sequence(from 5’to 3’)
6mer-oxoG1	5’-C (8-oxodG) CGCG-3’
6mer-oxoG2	5’-CGC (8-oxodG) CG-3’
6mer-3G	5’-CGCGCG-3’

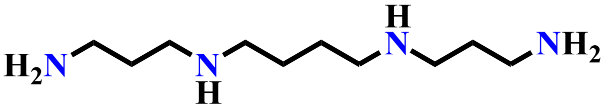

spermine (spm)

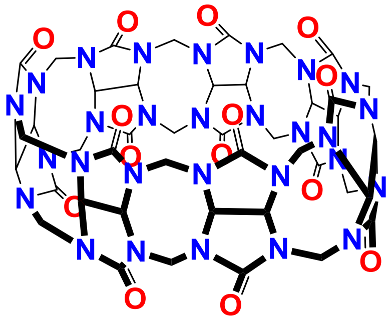

Cucurbit[7]uril (CB7)

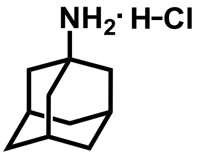

1-Adamantanamine hydrochloride (AM)

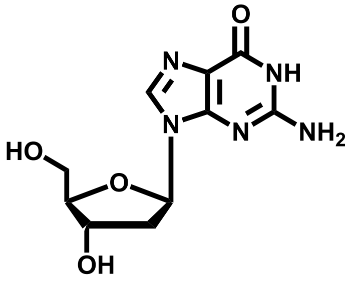

dG

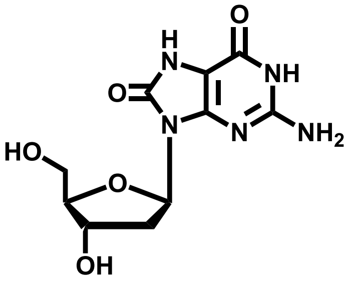

8-oxodG

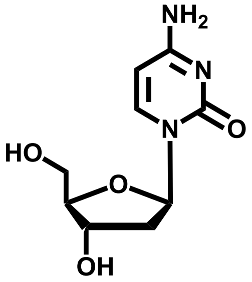

dC

Figure S1: Demonstration of chemical structures of spm, CB7, AM, dG, 8-oxodG and dC.

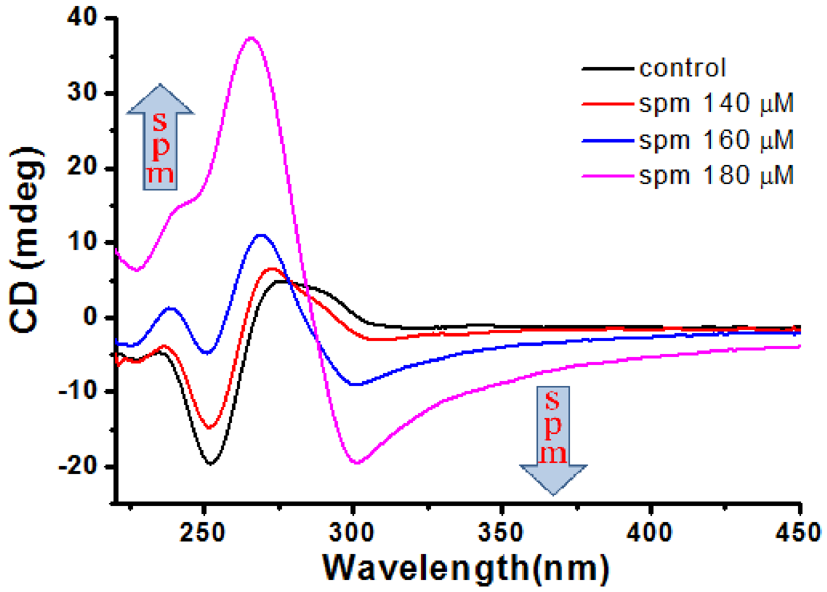

Figure S2: The influence of spm on DNA helical structure.
In this assay, the starting polyGC sample (150 µM) was heated to 90 °C for 5.0 min and then cooled slowly to room temperature. The above preparation was then exposed to increasing concentrations of spm at room temperature for 15 min. The sample was prepared in 10 mM Tris-HCl buffer at pH 7.0 with 50 mM NaCl. The arrows indicate the change in CD absorbance upon the addition of spm.

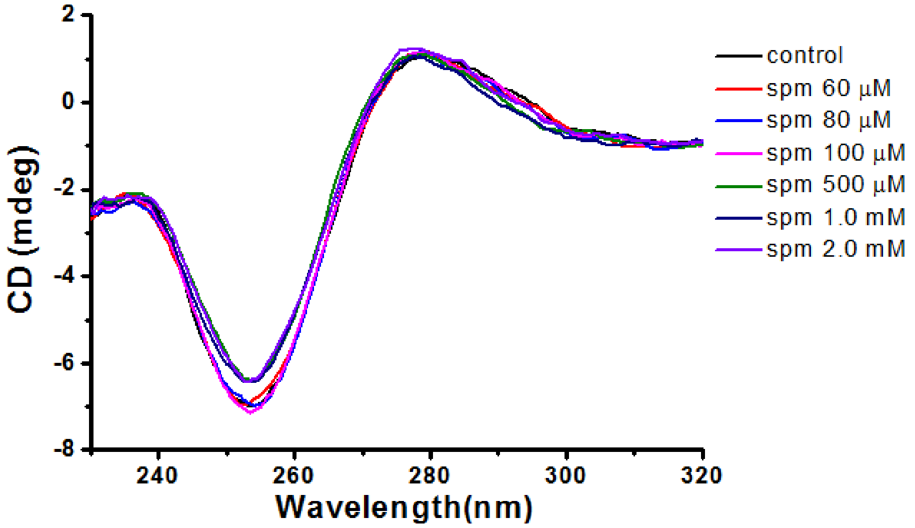

Figure S3: The influence of spm on DNA helical structure.
In this assay, the 6mer-3G (50 μM) was exposed to increasing concentrations of spm at room temperature for 15 min. The sample was prepared in 10 mM Tris-HCl buffer at pH 7.0 with 50 mM NaCl. The sequential addition of spm did not cause a significant change in the CD spectrum of 6mer-3G. Moreover, the 6mer-3G takes the B-form at spm concentrations up to 2.0 mM.

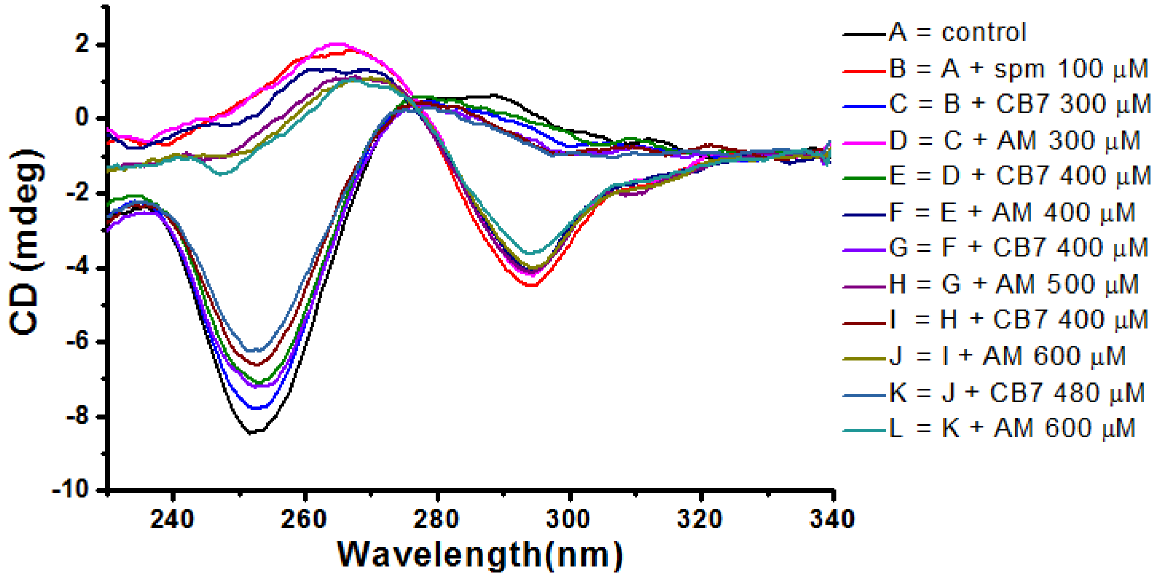

Figure S4: Repetitive switching of DNA helical structures between B-form and Z-form. In this demonstration, the black line indicates the CD spectrum of the 6mer-oxoG1 sample (50 μM) at room temperature. Subsequently, spm was added followed by an incubation at room temperature for 15 min, and the resultant solution was subjected to CD measurement (the red line). Followingly, CB7 and AM were sequentially added to the same sample as indicated followed by an incubation at room temperature for 15 min. After each addition, the sample was measured and the CD spectrum was shown in response to the input (the third to twelfth lines). The 6mer-oxoG1 sample was prepared in 10 mM Tris-HCl buffer at pH 7.0 with 50 mM NaCl. Conditions are given in the figure legend.

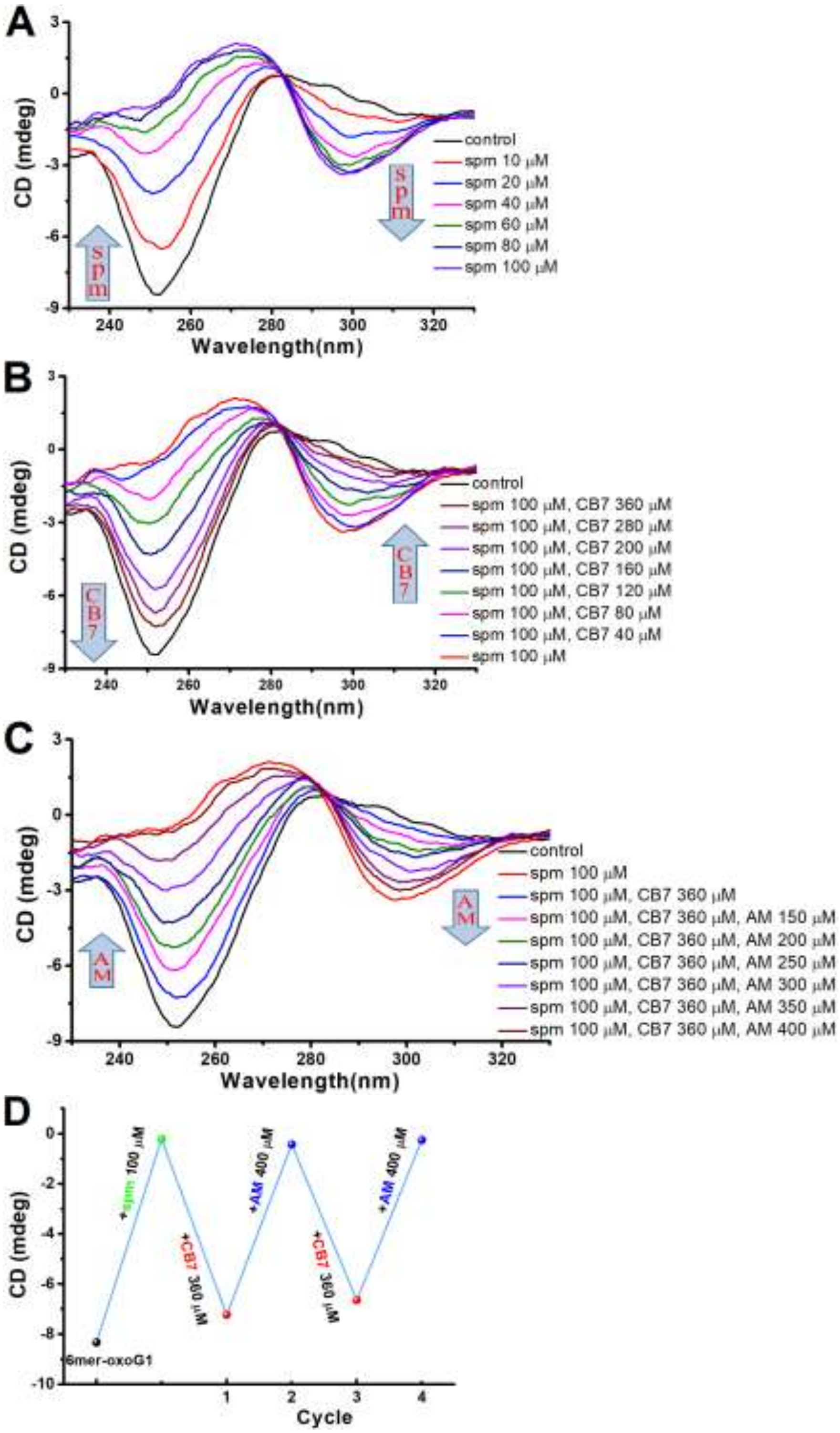

Figure S5: Supramolecular B/Z transition in a short oligonucleotide with 8-oxodG. (**A**) The influence of spm on DNA helical structures. In this assay, the 6mer-oxoG2 (50 µM) was exposed to spm at room temperature for 15 min. Upon addition of increasing amounts of spm, there was a gradual appearance of a negative peak at approximately 290 nm and a positive peak at about 260 nm. (**B**) The influence of CB7 on DNA helical structures. In this assay, the 6mer-oxoG2 (50 µM) was exposed to 100 µM spm, and then treated with increasing amounts of CB7 at room temperature for 15 min. Specifically, the treatment with 80 µM CB7 resulted in a significant decrease of Z-DNA formation, and this effect was more evident with the 160 and 280 µM treatments. (**C**) The influence of AM on DNA helical structures. In this assay, the starting sample (50 µM 6mer-oxoG2, 100 µM spm and 360 µM

CB7) was prepared and subjected to increasing concentrations of AM at room temperature for 15 min. Specifically, B- to Z-DNA transition was obviously enhanced by the 150 µM AM treatment, and higher levels of enhancement were observed with the 250 and 350 µM treatments. (**D**) Repetitive switching of helical structures between B-DNA and Z-DNA. In this demonstration, the starting point indicates the CD absorbance of a 50 µM 6mer-oxoG2 sample at room temperature. Subsequently, spm was added, and the resulting solution was subjected to CD measurement (second point). Next, CB7 and AM were sequentially added to the same sample as indicated. After each addition, the solution was measured, and CD absorbance was shown in response to the input (the third to sixth points). Conditions are given in the text. For (**A**), (**B**), (**C**) and (**D**), the 6mer-oxoG2 sample was prepared in 10 mM Tris-HCl buffer at pH 7.0 with 50 mM NaCl. The arrows indicate the change in CD absorbance upon the addition of spm, CB7 or AM.

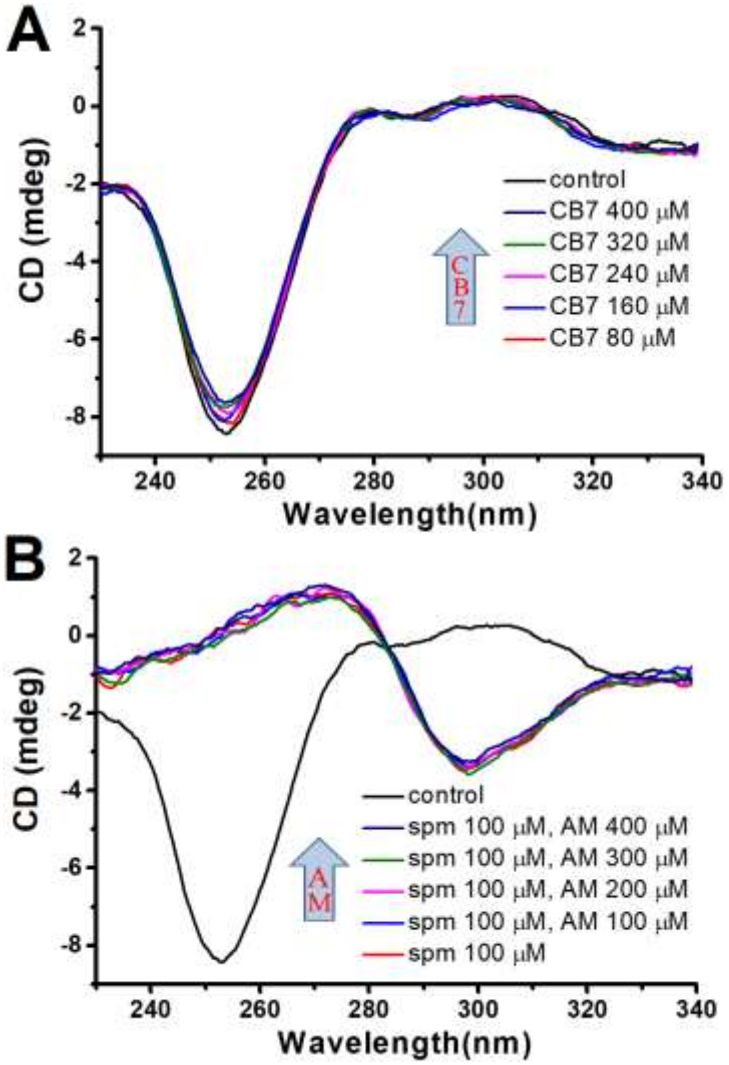

Figure S6: The influence of different treatments on DNA helical structure.
(**A**) The influence of CB7 on DNA helical structure. In this assay, the 6mer-oxoG1 (50 μM) was exposed to increasing concentrations of CB7 at room temperature for 15 min. (**B**) The influence of AM on DNA helical structure. In this assay, the 6mer-oxoG1 (50 μM) was exposed to 100 μM spm, and then treated with increasing amounts of AM at room temperature for 15 min before determination of the CD spectrum. For (**A**) and (**B**), the 6mer-oxoG1 sample was prepared in 10 mM Tris-HCl buffer at pH 7.0 with 50 mM NaCl. In itself, the CB7 or AM did not cause a significant change in the CD spectrum of 6mer-oxoG1.

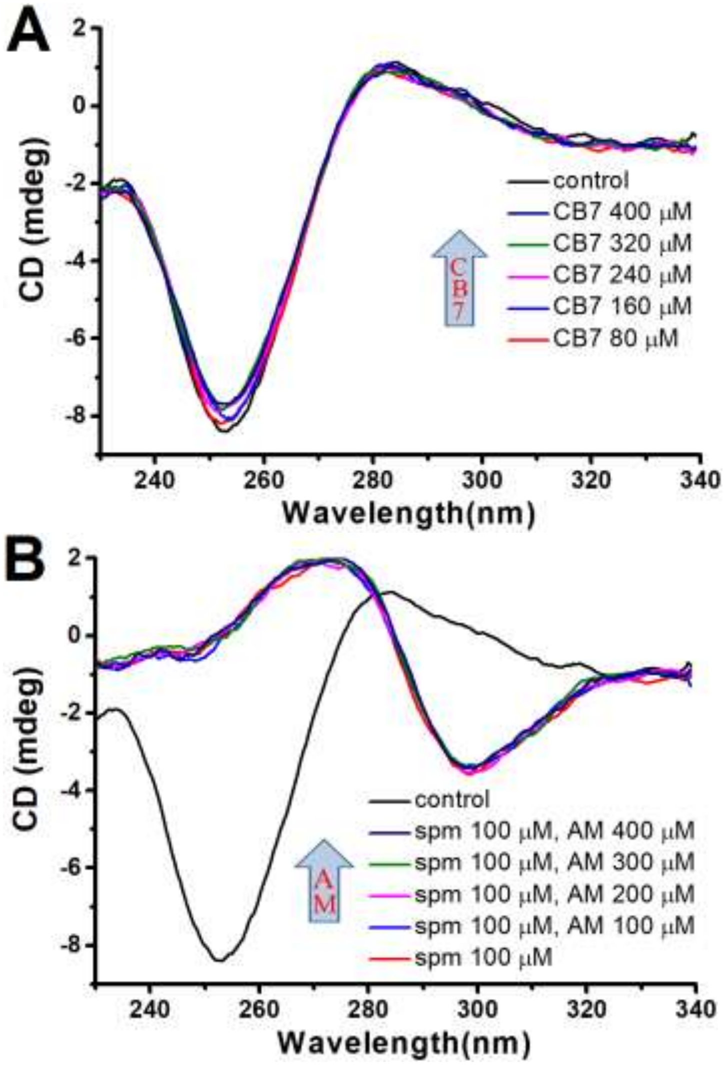

Figure S7: The influence of different treatments on DNA helical structure.
(**A**) The influence of CB7 on DNA helical structures. In this assay, the 6mer-oxoG2 (50 μM) was exposed to increasing concentrations of CB7 at room temperature for 15 min. (**B**) The influence of AM on DNA helical structures. In this assay, the 6mer-oxoG2 (50 μM) was exposed to 100 μM spm, and then treated with increasing amounts of AM at room temperature for 15 min before determination of the CD spectrum. For (**A**) and (**B**), the 6mer-oxoG2 sample was prepared in 10 mM Tris-HCl buffer at pH 7.0 with 50 mM NaCl. In itself, the CB7 or AM did not cause a significant change in the CD spectrum of 6mer-oxoG2.

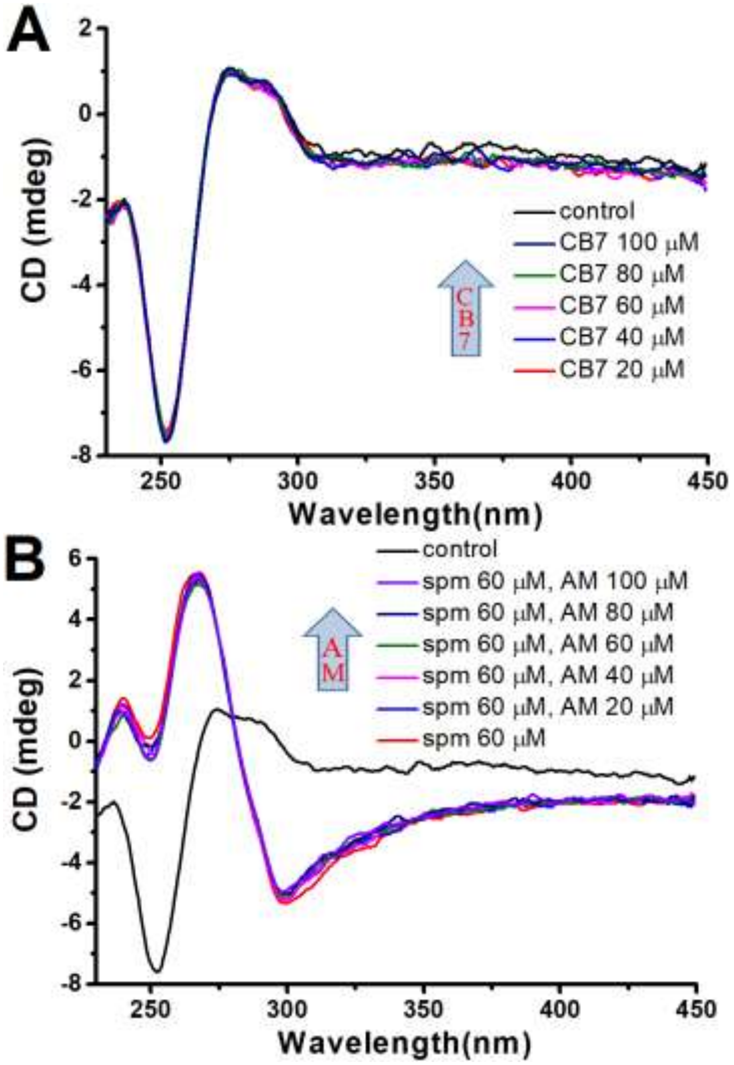

Figure S8: The influence of different treatments on DNA helical structure.
(**A**) The influence of CB7 on DNA helical structures. In this assay, the polyGC (150 μM) was exposed to increasing concentrations of CB7 at room temperature for 15 min. (**B**) The influence of AM on DNA helical structures. In this assay, the polyGC (150 μM) was exposed to 100 μM spm, and then treated with increasing amounts of AM at room temperature for 15 min before determination of the CD spectrum. For (**A**) and (**B**), the polyGC sample was prepared in 10 mM Tris-HCl buffer at pH 7.0 with 50 mM NaCl. In itself, the CB7 or AM did not cause a significant change in the CD spectrum of polyGC.

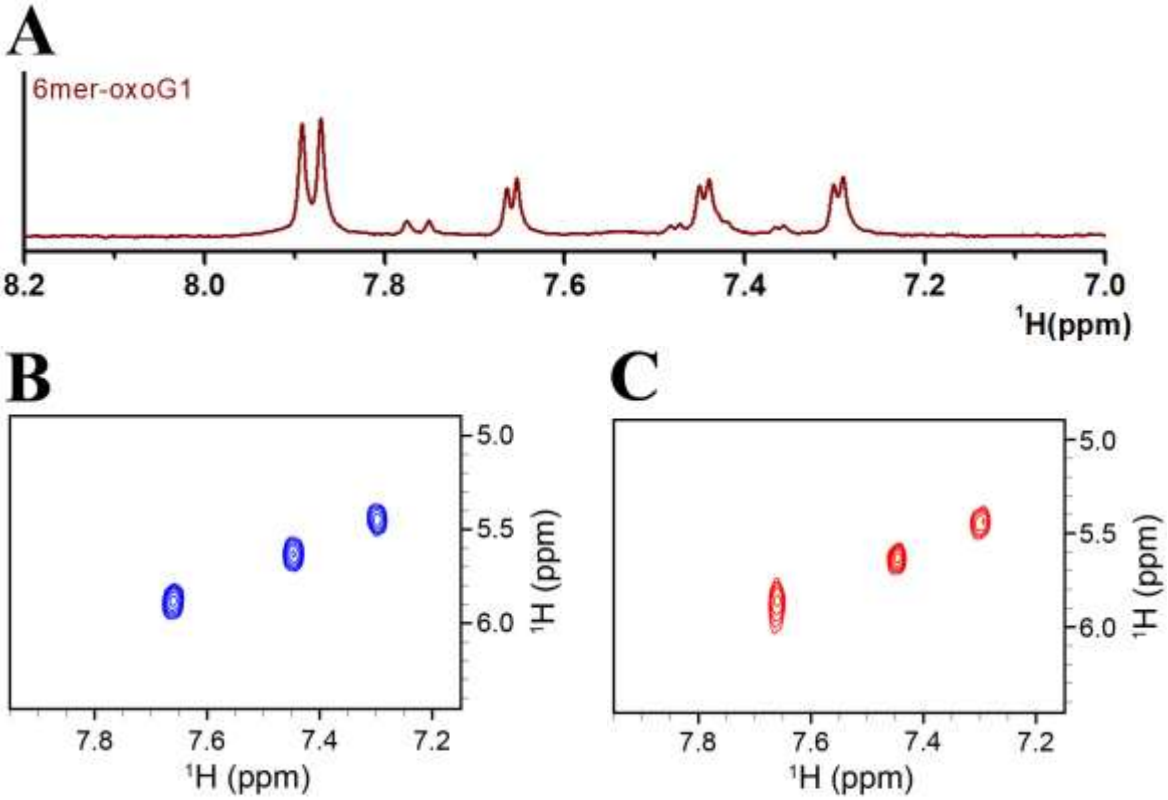

Figure S9: ^{1}H-NMR refinement of DNA conformation in the absence of spm.
(**A**) The 1D ^{1}H-NMR spectrum of 6mer-oxoG1 in selected region is demonstrated. (**B**) The 2D COSY spectrum of 6mer-oxoG1 in selected region is demonstrated. (**C**) The 150-ms NOESY spectrum of 6mer-oxoG1 in selected region is demonstrated. For (**A**), (**B**) and (**C**), the oligonucleotide 6mer-oxoG1 (200 μM) was used. The spectra were acquired at 298 K in a buffered solution (2 mM Tris-HCl, 50 mm NaCl, 80% D_2O/20% H_2O, pH 7.0). The B-form of 6mer-oxoG1 is indicated by the absence of NOE cross-peak between dG H8 and deoxyribose H1'.

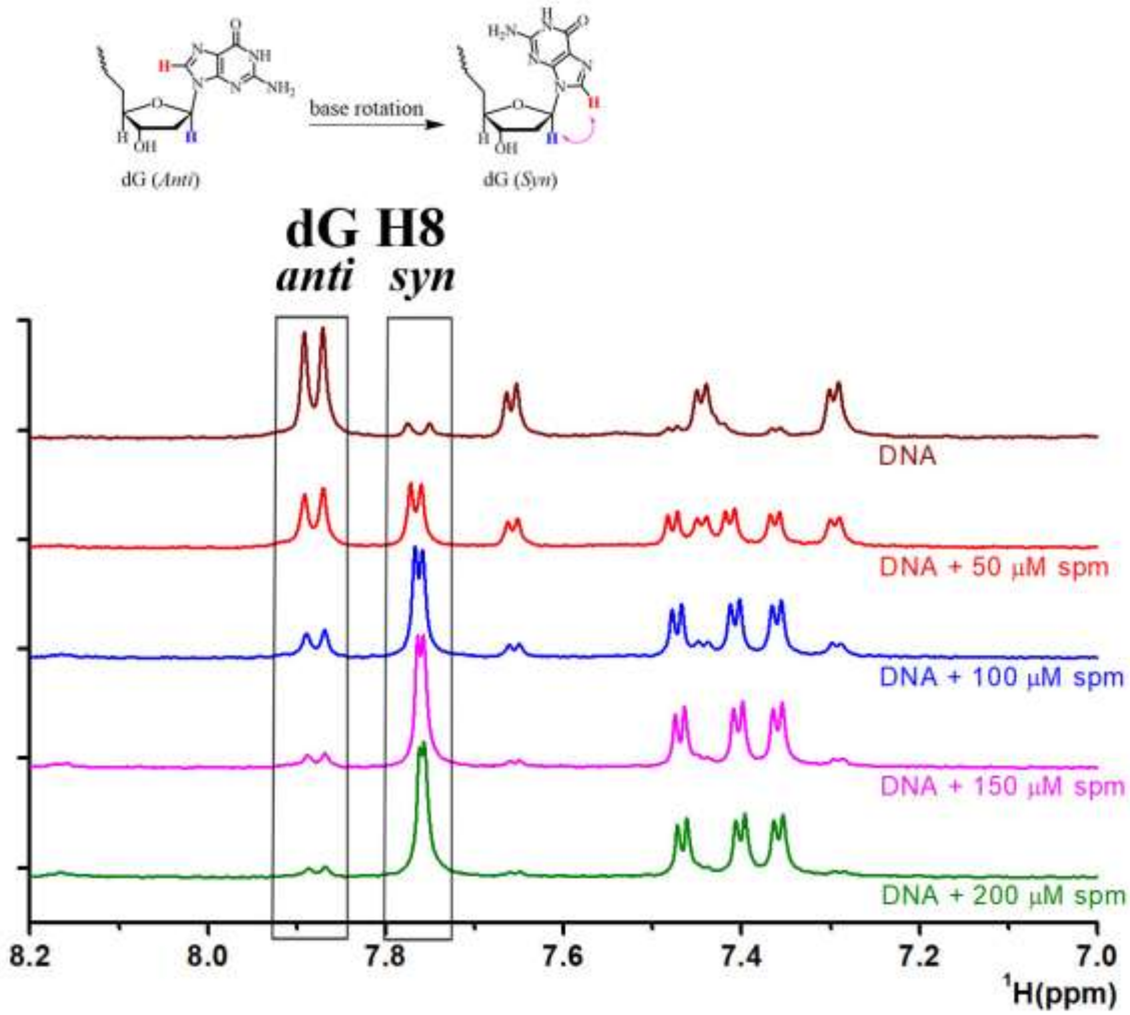

Figure S10: 1D ^{1}H-NMR titration of 6mer-oxoG1 with spm.
In this assay, the 6mer-oxoG1 (200 μM) was treated with different concentrations of spm at room temperature for 15 min. The 1D ^{1}H-NMR spectra were acquired at 298 K in a buffered solution (2 mM Tris-HCl, 50 mm NaCl, 80% D_2O/20% H_2O, pH 7.0). The chemical shifts of dG H8 were observed to gradually shift upfield with increasing amounts of spm.

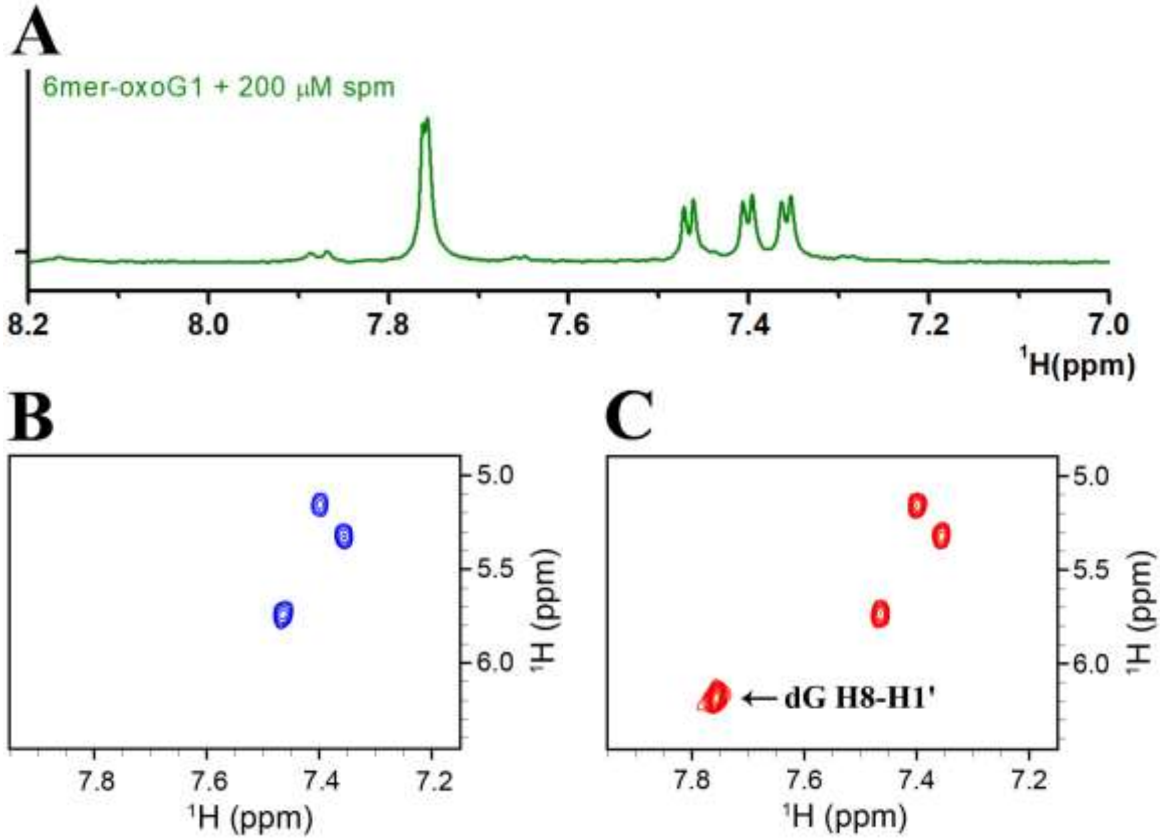

Figure S11: ^{1}H-NMR refinement of DNA conformation in the presence of 200 μM spm. (**A**) The 1D ^{1}H-NMR spectrum in selected region was demonstrated. (**B**) The 2D COSY spectrum in selected region was demonstrated. (**C**) The 150-ms NOESY spectrum in selected region demonstrated dG H8-H1' cross-peak. For (**A**), (**B**) and (**C**), the oligonucleotide 6mer-oxoG1 (200 μM) was used. The spectra were acquired at 298 K in a buffered solution (2 mM Tris-HCl, 50 mm NaCl, 80% D_2O/20% H_2O, pH 7.0). The Z-form of 6mer-oxoG1 is indicated by the presence of NOE cross-peak between dG H8 and deoxyribose H1'.

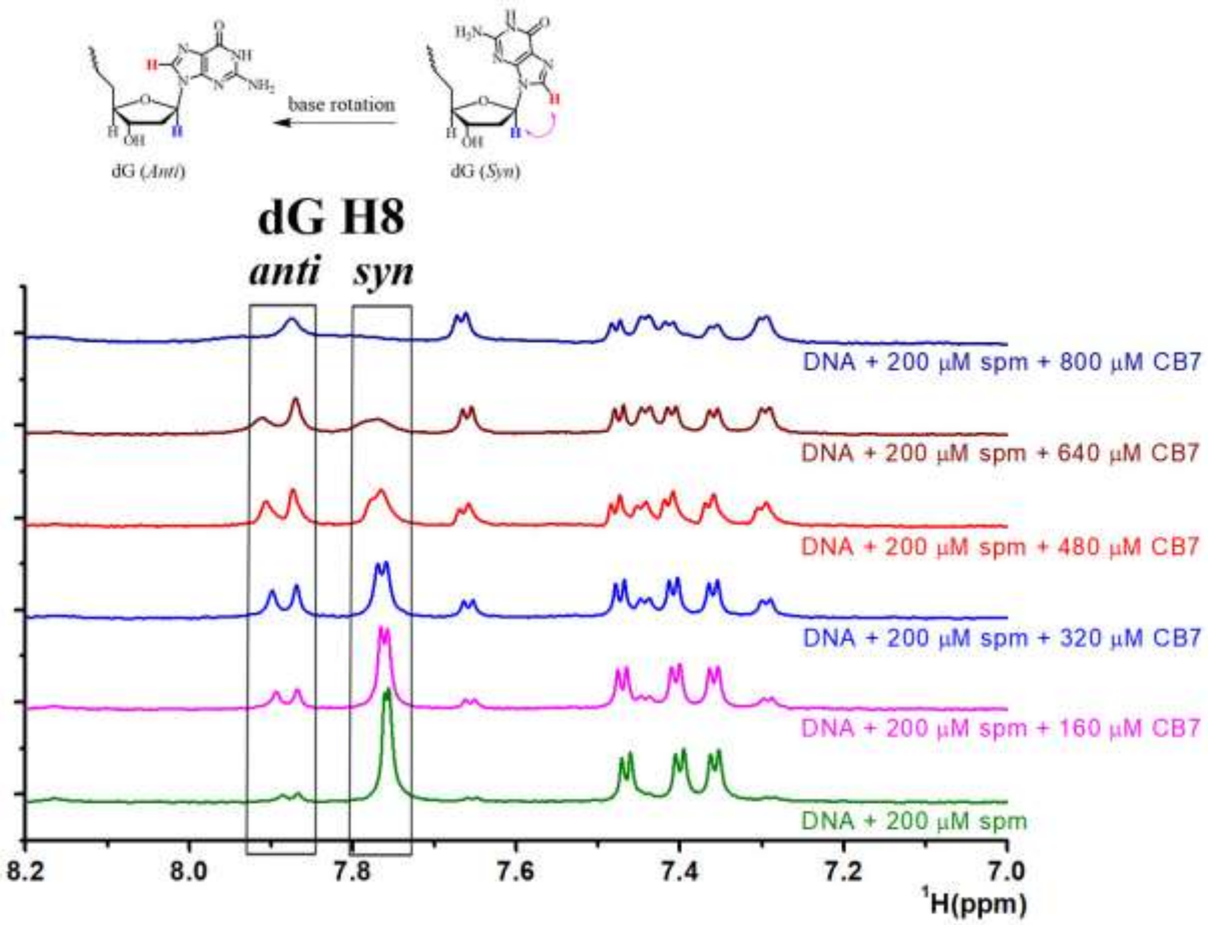

Figure S12: 1D ^{1}H-NMR titration of 6mer-oxoG1 with CB7.
In this assay, the 6mer-oxoG1 (200 µM) was exposed to 200 µM spm, and then treated with increasing amounts of CB7 at room temperature for 15 min. The 1D ^{1}H-NMR spectra were acquired at 298 K in a buffered solution (2 mM Tris-HCl, 50 mm NaCl, 80% D_2O/20% H_2O, pH 7.0). The chemical shifts of dG H8 were observed to gradually shift downfield with increasing amounts of spm.

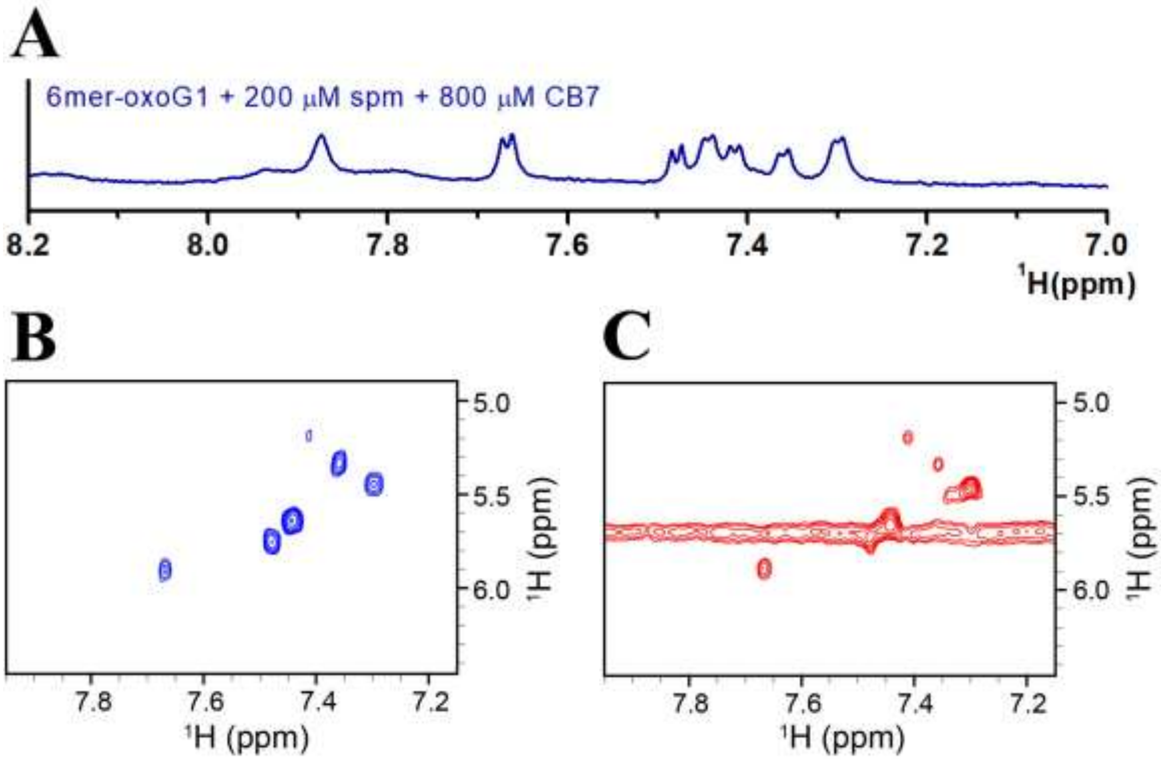

Figure S13: ^{1}H-NMR refinement of DNA conformation in the presence of 200 µM spm and 800 µM CB7.
(**A**) The 1D ^{1}H-NMR spectrum in selected region was demonstrated. (**B**) The 2D COSY spectrum in selected region was demonstrated. (**C**) The 150-ms NOESY spectrum in selected region was demonstrated. For (**A**), (**B**) and (**C**), the oligonucleotide 6mer-oxoG1 (200 µM) was used. The spectra were acquired at 298 K in a buffered solution (2 mM Tris-HCl, 50 mm NaCl, 80% D_2O/20% H_2O, pH 7.0). The B-form of 6mer-oxoG1 is indicated by the absence of NOE cross-peak between dG H8 and deoxyribose H1'.

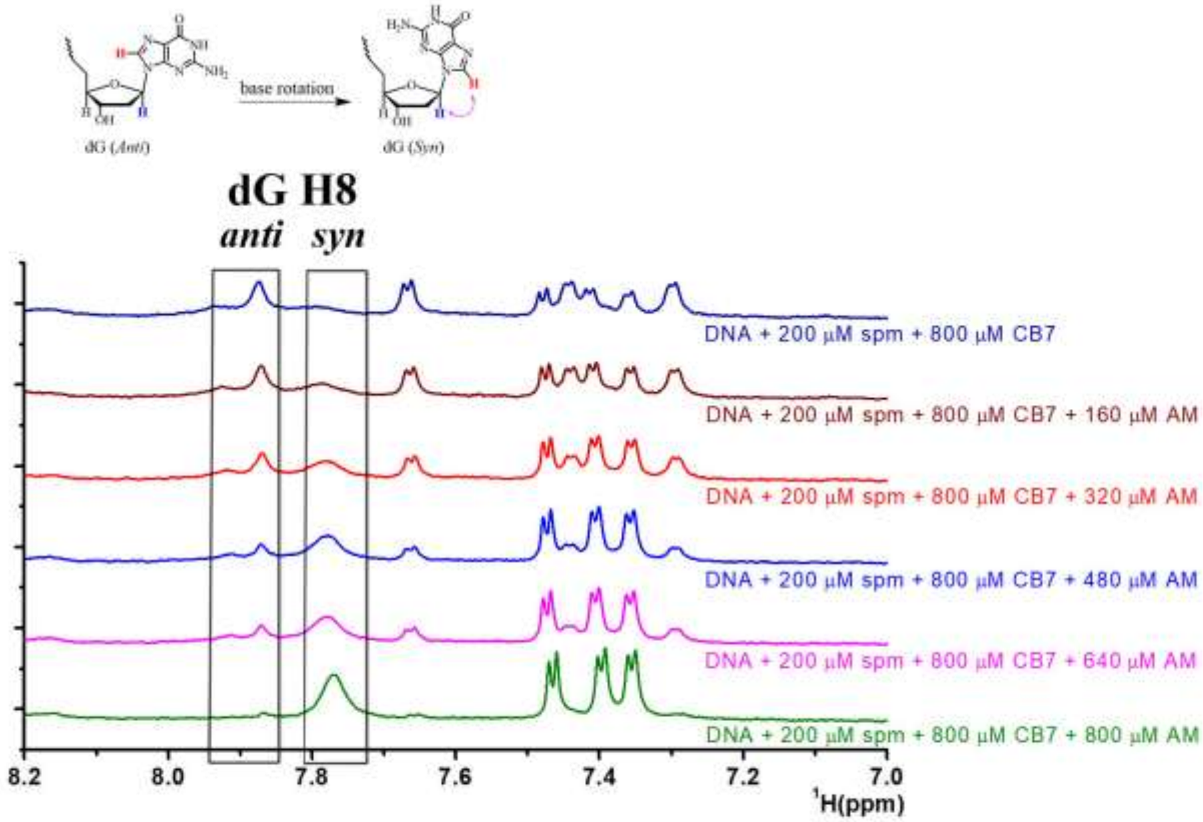

Figure S14: 1D ^{1}H-NMR titration of 6mer-oxoG1 with AM.
In this assay, the starting sample (200 µM 6mer-oxoG1, 200 µM spm and 800 µM CB7) was prepared and subjected to increasing concentrations of AM at room temperature for 15 min. The 1D ^{1}H-NMR spectra were acquired at 298 K in a buffered solution (2 mM Tris-HCl, 50 mm NaCl, 80% D_2O/20% H_2O, pH 7.0). The chemical shifts of dG H8 were observed to gradually shift downfield with increasing amounts of AM.

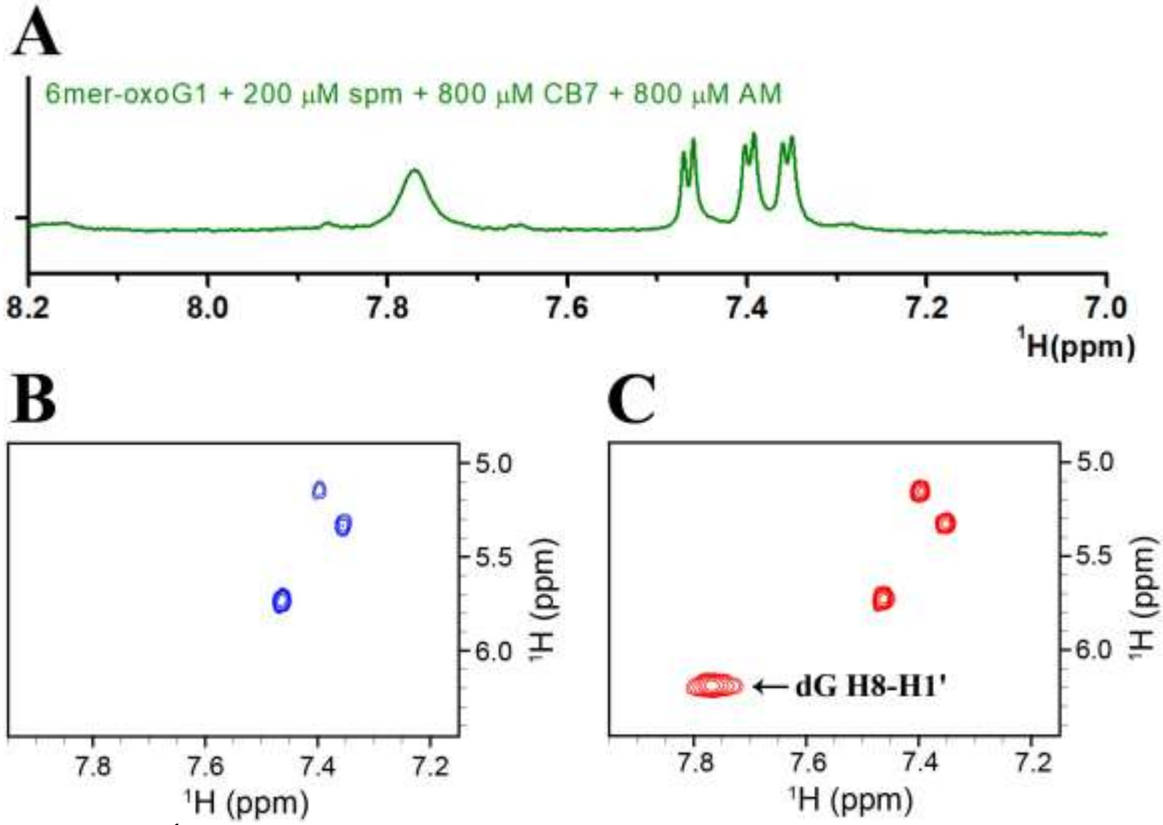

Figure S15: ^{1}H-NMR refinement of DNA conformation in the presence of spm, CB7 and AM.
(**A**) The 1D ^{1}H-NMR spectrum in selected region was demonstrated. (**B**) The 2D COSY spectrum in selected region was demonstrated. (**C**) The 150-ms NOESY spectrum in selected region demonstrated dG H8-H1' cross-peak. For (**A**), (**B**) and (**C**), the oligonucleotide 6mer-oxoG1 (200 μM) was used. The spectra were acquired at 298 K in a buffered solution (2 mM Tris-HCl, 50 mm NaCl, 80% D_2O/20% H_2O, pH 7.0). The Z-form of 6mer-oxoG1 is indicated by the presence of NOE cross-peak between dG H8 and deoxyribose H1'.

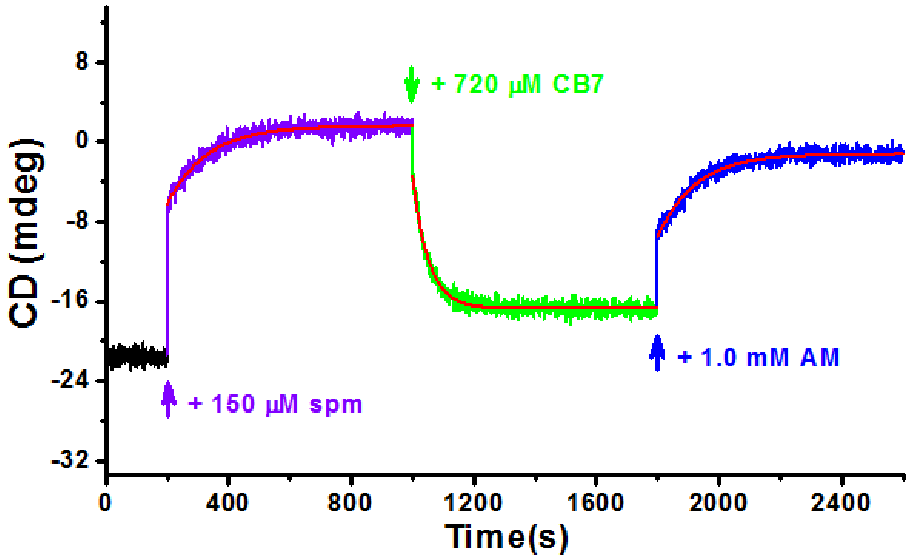

Figure S16: The B/Z-DNA transition kinetics.
This study monitored the time course of CD intensity (253 nm) change of 6mer-oxoG1 (120 μM) (data points in different colors). Red lines are mono-exponential fits of the according experimental data. In the first experiment, spm was added to induce B-Z transition of 6mer-oxoG1. As indicated, this transition occurs very fast (purple data points). The CD ellipticity at 253 nm shows a rapid jump followed by a rise which plateaus in a period less than 400 s. The kinetics curve can be well fitted to the mono-exponential function (red fitted line), suggesting that the spm-induced transition from B- to Z-DNA follows the first order. In the second experiment, CB7 was added to reverse the conformation of 6mer-oxoG1. Following the addition of CB7, a rapid decrease of absorbance was observed due to DNA conformational change (green data points). This time-dependent change follows single exponential decay (red fitted line), also indicating the first-order kinetics. Moreover, after the addition of AM, the absorbance increases logarithmically due to the B- to Z-DNA transition (blue data points and red fitted line).

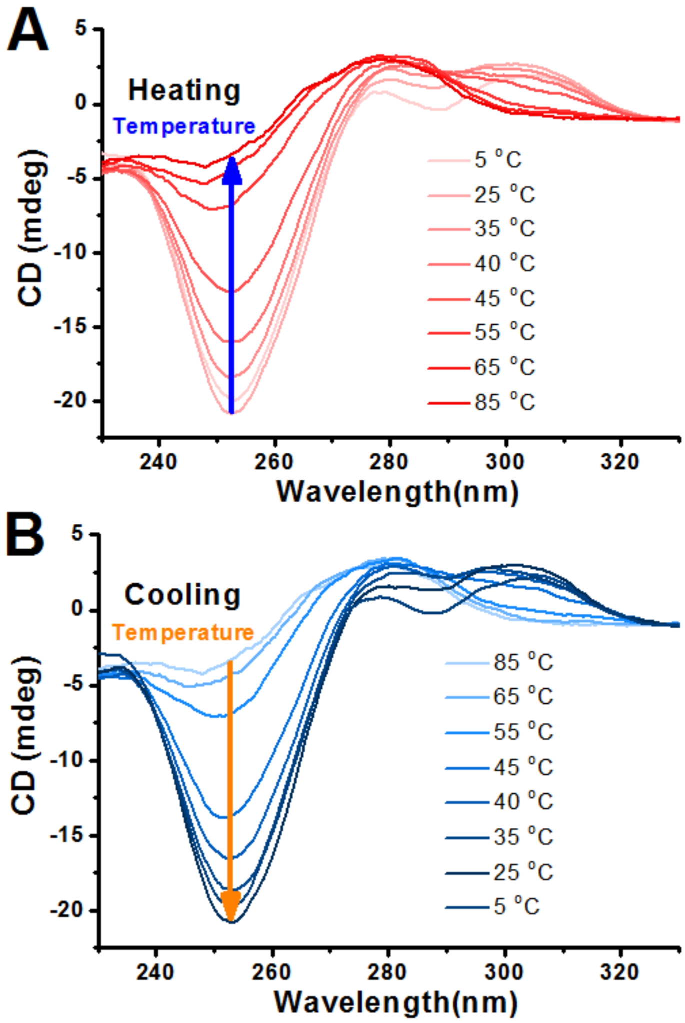

Figure S17: Thermal denaturation and renaturation of B-DNA in the absence of spm. (**A**) The CD spectra of 6mer-oxoG1 as a function of increasing temperatures. (**B**) The CD spectra of 6mer-oxoG1 as a function of decreasing temperatures. For (**A**) and (**B**), the temperatures (from 5 °C to 85 °C) at which the CD spectra were collected are indicated in the figure legend. The arrow indicates direction of change in the CD spectra upon varied temperatures. The 6mer-oxoG1 (120 μM) was prepared in 10 mM Tris-HCl buffer (pH 7.0, 50 mM NaCl). The sample was incubated at each specified temperature for 15 min prior to recording of the CD spectra. When the spm is absent, heating and cooling experiments showed that thermal denaturation and renaturation of the original B-DNA are reversible.

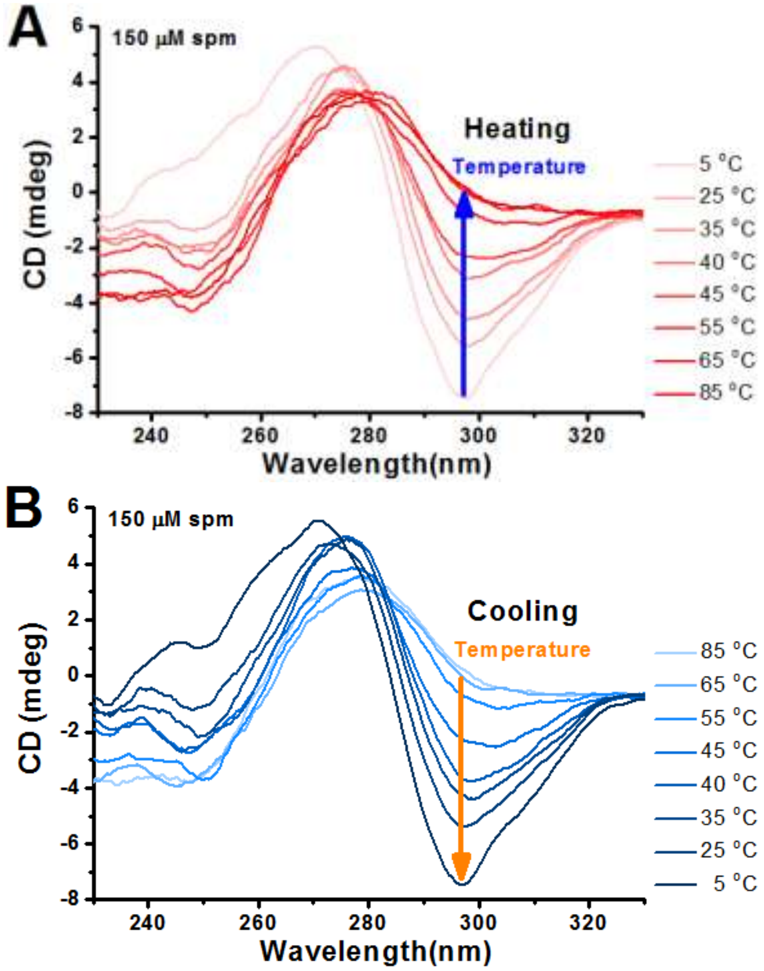

Figure S18: Thermal denaturation and renaturation of Z-DNA in the presence of spm. (**A**) The CD spectra of 6mer-oxoG1 as a function of increasing temperatures. (**B**) The CD spectra of 6mer-oxoG1 as a function of decreasing temperatures. For (**A**) and (**B**), the temperatures (from 5 °C to 85 °C) at which the CD spectra were collected are indicated in the figure legend. The arrow indicates direction of change in the CD spectra upon varied temperatures. The 6mer-oxoG1 (120 μM) was prepared in 10 mM Tris-HCl buffer (pH 7.0, 50 mM NaCl) with 150 μM spm. The sample was incubated at each specified temperature for 15 min prior to recording of the CD spectra. In the presence of spm, the resulted Z-DNA appears to melt directly into single strands rather than transforming into other helical structures prior to melting. By decreasing the temperature, renaturation of the heat-denatured DNA into the Z-form was observed.

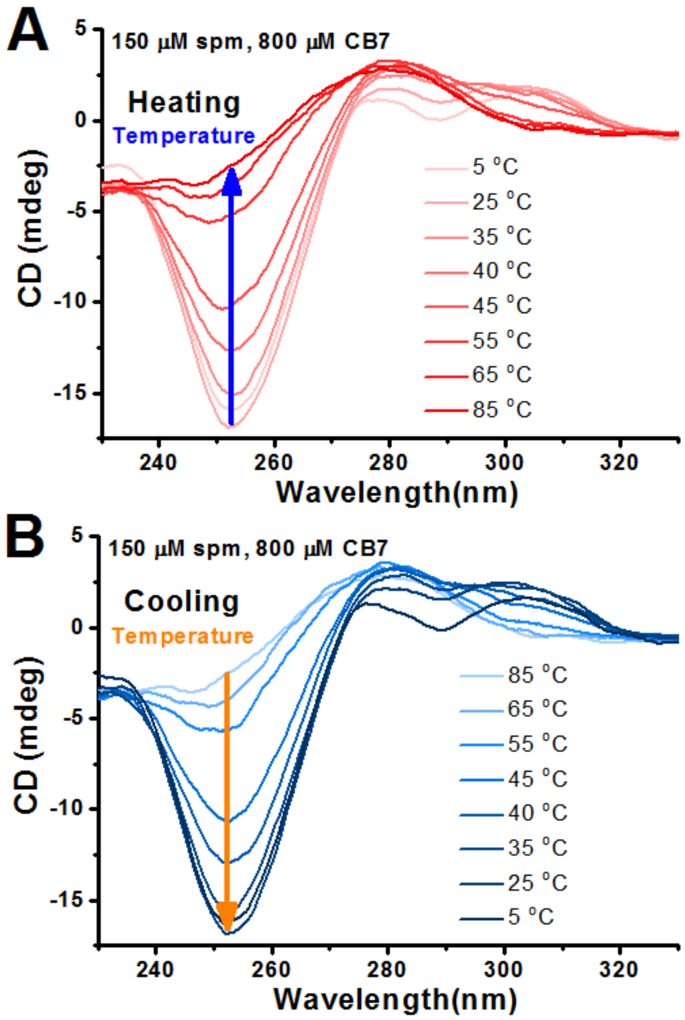

Figure S19: Thermal denaturation and renaturation of B-DNA in the presence of spm and CB7.

(**A**) The CD spectra of 6mer-oxoG1 as a function of increasing temperatures. (**B**) The CD spectra of 6mer-oxoG1 as a function of decreasing temperatures. For (**A**) and (**B**), the temperatures (from 5 °C to 85 °C) at which the CD spectra were collected are indicated in the figure legend. The arrow indicates direction of change in the CD spectra upon varied temperatures. The 6mer-oxoG1 (120 μM) was prepared in 10 mM Tris-HCl buffer (pH 7.0, 50 mM NaCl) with 150 μM spm and 800 μM CB7. The sample was incubated at each specified temperature for 15 min prior to recording of the CD spectra. In the presence of spm and CB7, the resulting B-form is thermally stable, with direct melting into single strands. When the temperature was reduced, again it went back to the B-form along a reversible way.

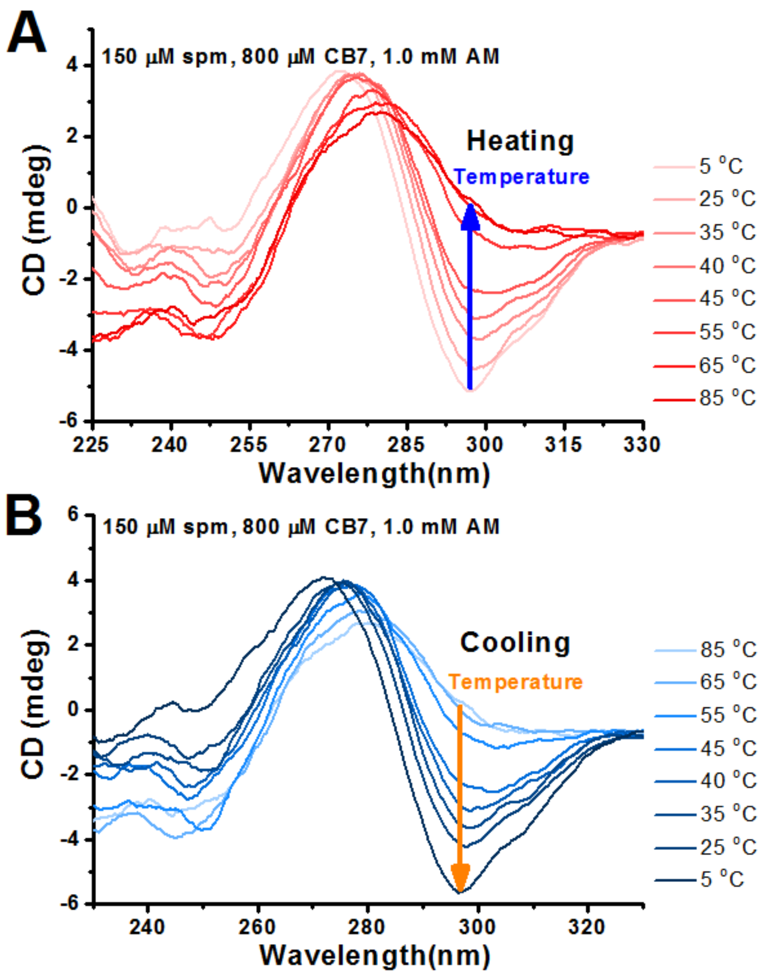

Figure S20: Denaturation and renaturation of Z-DNA in the presence of spm, CB7 and AM.
(**A**) The CD spectra of 6mer-oxoG1 as a function of increasing temperatures. (**B**) The CD spectra of 6mer-oxoG1 as a function of decreasing temperatures. For (**A**) and (**B**), the temperatures (from 5 °C to 85 °C) at which the CD spectra were collected are indicated in the figure legend. The arrow indicates direction of change in the CD spectra upon varied temperatures. The 6mer-oxoG1 (120 µM) was prepared in 10 mM Tris-HCl buffer (pH 7.0, 50 mM NaCl) with 150 µM spm, 800 µM CB7 and 1.0 mM AM. The sample was incubated at each specified temperature for 15 min prior to recording of the CD spectra. In the presence of spm, CB7 and AM, no apparent conformational transition, other than denaturation into single strands, has been observed upon heating. By decreasing the temperature, the single-stranded DNA appears to refold into the Z-form.

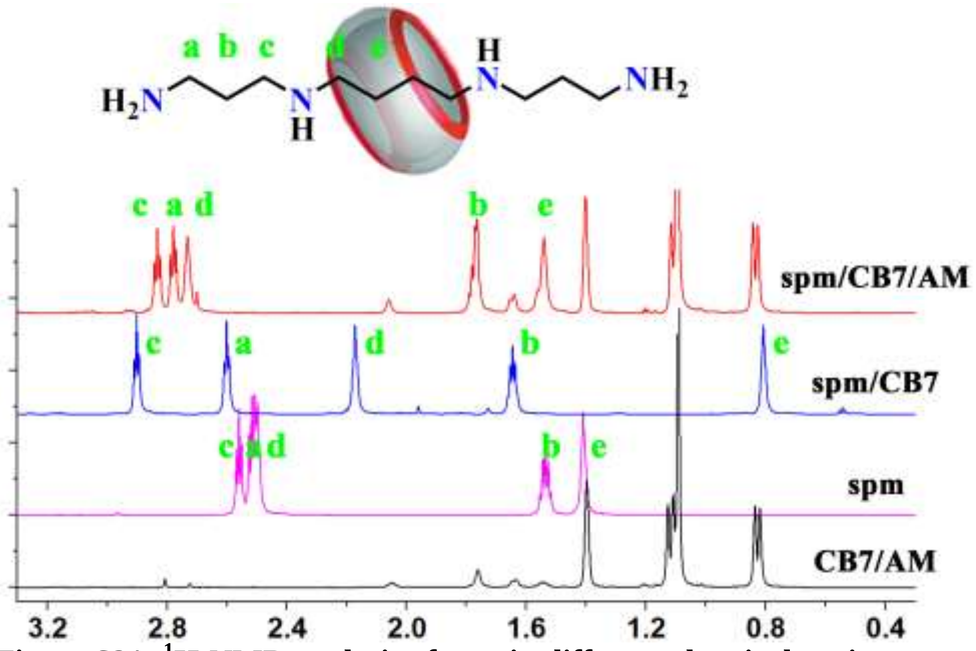

Figure S21: ^{1}H-NMR analysis of spm in different chemical environments.
The spectrum overlay of the selected region (δ = 3.2 - 0.4 ppm) is demonstrated. All methylene groups of spm are labelled by green letters (a - e) in the chemical structure.

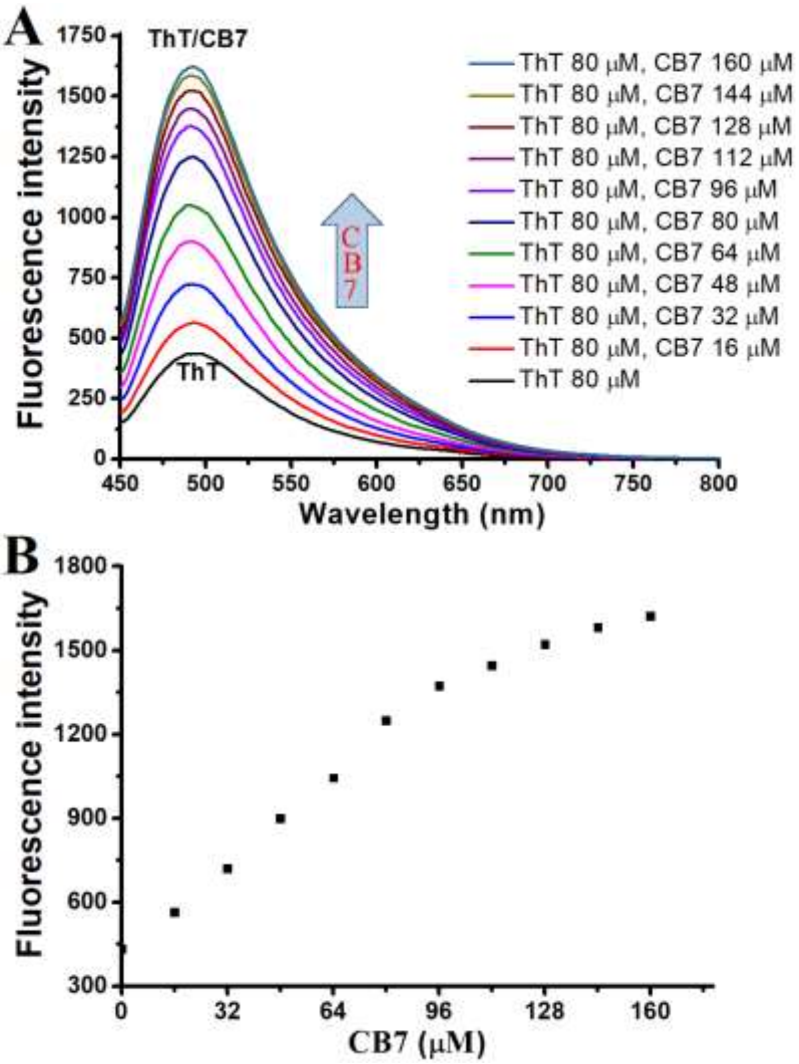

Figure S22: Fluorescence titrations of ThT by CB7 in aqueous solution.
(**A**) The corresponding fluorescence spectral changes of ThT (80 μM) upon CB7 treatment are demonstrated. (**B**) The fluorescence of ThT at 493 nm was plotted against the concentrations of added CB7 (0 - 160 μM). For this assay, ThT (80 μM) was dissolved in the desired buffer (10 mM Tris-HCl at pH 7.0, 50 mM NaCl) and the fluorescence spectrum of this sample was determined. After that, increasing amounts of CB7 were added sequentially with fast mixing. The fluorescent emission spectra were then determined at room temperature using a LS55 fluorescence spectrometer (Perkin-Elmer Inc., USA). and the samples were incubated for 15 min before determination of the fluorescence spectrum.

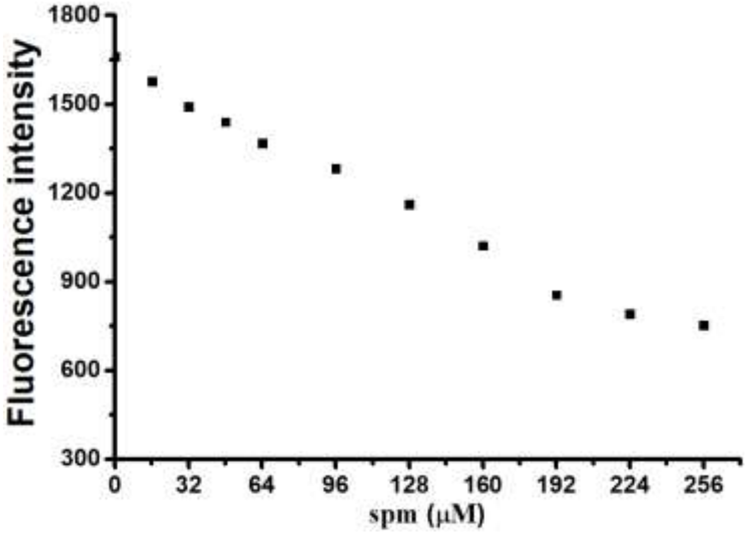

Figure S23: Fluorescence titrations of the CB7/ThT complex by spm in aqueous solution. The fluorescence of the CB7/ThT complex at 493 nm was plotted against the concentrations of added spm (0 - 256 μM). The CB7/ThT complex (80 μM, CB7:ThT = 2:1) was prepared in the desired buffer (10 mM Tris-HCl at pH 7.0, 50 mM NaCl) and the fluorescence spectrum of this sample was determined. Subsequently, increasing amounts of spm (from 16 to 256 μM) were added sequentially with fast mixing. The fluorescent emission spectra were then determined at room temperature using a LS55 fluorescence spectrometer (Perkin-Elmer Inc., USA).
